# Recent Insights About Probiotics Related Pharmabiotics in Pharmacology: Prevention and Management of Diseases

**DOI:** 10.1007/s12602-025-10613-3

**Published:** 2025-06-23

**Authors:** Sena Davran Bulut, H. Ali Döndaş, Hasan Ufuk Celebioglu, José M. Sansano, Naciye Yaktubay Döndaş

**Affiliations:** 1 Department of Biotechnology, Institute of Natural and Applied Sciences, Çukurova University, Balcalı, Adana, 01330 Turkey; 2 Department of Basic Pharmaceutical Sciences, Faculty of Pharmacy, Çukurova University, Balcalı, Adana, 01330 Turkey; 3 Department of Biotechnology, Faculty of Science, Bartin University, Bartin, 74100 Turkey; 4 Department of Organic Chemistry, Research National Center ORFEO-CINQA and Institute of Organic Synthesis, University of Alicante, Alicante, 03690 Spain; 5 Department of Medical Pharmacology, Faculty of Medicine, Cukurova University, Adana, 01330 Turkey; 6 Department of Translational Medicine, Institute of Health Sciences, Çukurova University, Adana, 01330 Turkey

**Keywords:** Probiotics, Prebiotics, Synbiotics, Postbiotics, Paraprobiotics, Metabiotics, Next-generation probiotics, Pharmabiotics, Fecal microbiota transplantation

## Abstract

The science of pharmacology investigates the effects of drugs on living organisms and vice versa. The frequency of side effects of some drugs used in traditional pharmacological treatment approaches and/or their inability to provide adequate treatment has led to the importance of new drug research and development (R&D) studies. Recently, due to the discovery that some diseases are associated with an imbalanced microbiota (dysbiosis), there has been a surge of interest in therapeutic approaches that can restore balance (biosis) to the microbiota. This review discusses the current status of the pharmabiotic potential of probiotics, prebiotics, synbiotics, paraprobiotics, postbiotics, metabiotics, next-generation probiotics, and fecal microbiota transplantation; describes their pharmacological functions from gastrointestinal disorders to neurodegenerative diseases; and also discusses the developments in pharmaceutical applications of probiotics and their derivatives.

## Introduction

**Fig. 1:**
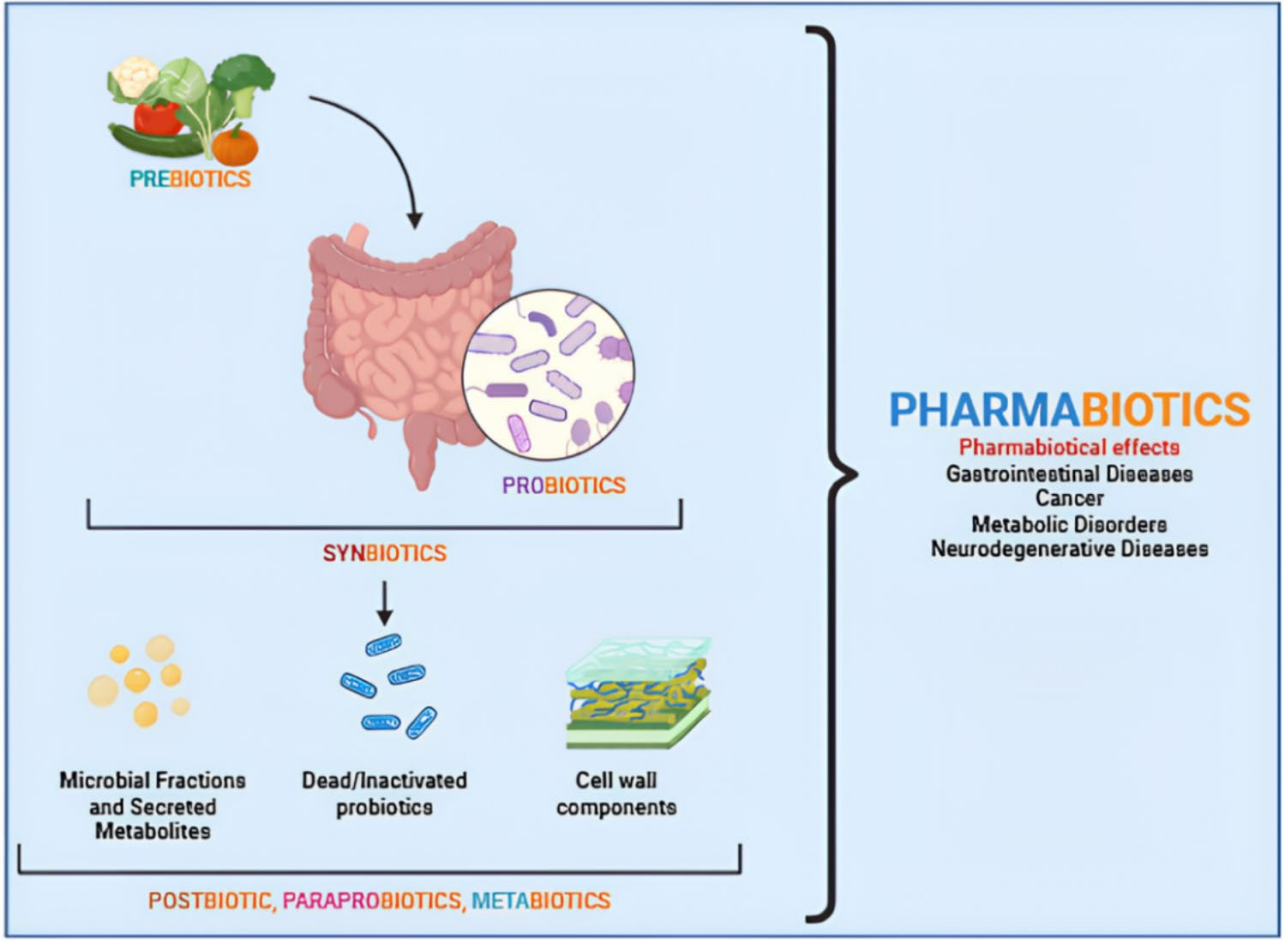
General representation of probiotics and related terms (generated using biorender.com)

Pharmacology focuses on the interaction between bioactive substances and the living organism (Fig. 1). Due to the frequency of side effects of drugs used in traditional treatment approaches and inadequate treatment, new drug R&D studies are becoming increasingly important [1]. Interest in probiotics has increased as recent R&D studies show that there is a connection between the microbiota in the body and diseases and that probiotics support human health through the microbiota [2]. The pharmaceutical industry’s interest in probiotics is also increasing due to the advantages of probiotic therapy, such as being more natural than conventional treatment approaches, having less side effect potential, and providing benefits for complex diseases [3]. Besides the administration of probiotics in the treatment of diseases, prebiotics, synbiotics, paraprobiotics, postbiotics, metabiotics, and next-generation probiotics have also been shown to be beneficial in providing microbiota biosis [4].

Prebiotics, synbiotics, probiotics, and their derivatives (paraprobiotics, postbiotics, metabiotics or next-generation probiotics) that can exert pharmacological effects through interaction with the host microbiota are called pharmabiotics [5]. Recently, there have been studies emphasizing the link between intestinal and/or extra-intestinal microbiota and achieving success in treatment with pharmabiotics [6, 7]. In a study by Wang et al. [8], the therapeutic effects of probiotics in chronic lipid metabolism disease were well explained through their pharmacological mechanisms of action. In the related study [8], safe probiotics isolated from nature were reported to be recognized as a novel therapeutic approach for the prevention and/or amelioration of chronic diseases. It was shown that probiotics regulate lipid synthesis by activating the AMP (5′adenosine monophosphate)-activated protein kinase (AMPK) signaling pathway and strengthen the intestinal barrier function by balancing the microbiota. It was also shown that the gut-liver axis that regulates gut flora metabolites such as short-chain fatty acids and bile acids was strengthened, and diet-induced lipid metabolism disorders were effectively ameliorated through the HMGCR/FXR/SHP signaling pathway [8]. Today, traditional treatment approaches for chronic diseases such as type 2 diabetes and hypertension, which are difficult to treat, use hypoglycemic and antihypertensive drugs, respectively, and since this treatment approach provides symptomatic treatment rather than radical treatment, the relevant traditional treatment may need to be lifelong [8–10]. Therefore, there is an increasing interest in alternative treatment approaches for the treatment of such chronic diseases.

Probiotics, which are commonly applied in infant formulas, fermented dairy products, and nutritional supplements, have been reported to contribute significantly to the treatment of various chronic diseases such as gastrointestinal diseases [11], cancer [12], and cardiovascular diseases [13]. Prebiotics are defined as the nondigestible food ingredients that beneficially affect the host health by selectively stimulating the growth of beneficial bacteria in the colon. Some prebiotics are complex carbohydrates that cannot be digested by humans and are used as a food source by beneficial microorganisms in the intestines [14]. In addition to carbohydrates, other types of compounds such as some (poly)phenols are suggested as having prebiotic potential because of their beneficial effects on host health and interaction with the microbiota. Prebiotics are derived from natural sources such as legumes and grains, as well as specific substances like inulin-type fructans and galacto-oligosaccharides [15].

It has been reported that their combined use (probiotics + prebiotics = synbiotics) creates synergism in effect compared to use alone [16]. This combination supports the growth of beneficial microbiota, maintains intestinal integrity, and prevents pathogen proliferation [17]. Postbiotics are metabolites of probiotics or components resulting from probiotic activities such as fermentation in the gut. Vitamins B and K, amino acids, and antimicrobial peptides synthesized by the intestinal microbiota are examples of postbiotics. Other types of postbiotics include lipopolysaccharides (LPS), enzymes, short-chain fatty acids, bacterial lysates, and cell-free supernatants [18]. Paraprobiotics, which have emerged recently and provide various health benefits to the host such as immunomodulatory, anti-inflammatory, antioxidant, and antihypertensive effects, are defined as “lifeless, dead, inactive or ghost probiotics” [19]. Additionally, paraprobiotics include non-viable microbial cells, microbial fractions, cellular components and bacterial lysates, peptidoglycans, polysaccharides from microbial cell walls, cell surface proteins, and teichoic acid, which improve host health through interactions with the gut microbiome [20]. Another important concept is the metabiotics, which are microbial low molecular weight compounds that can be used as drugs, bioactive food additives, or as enriching microbial components of functional foods to replenish the gut microbiota and improve health [21]. They include biologically active metabolites, signaling molecules, and cells (dead microbial cells and their parts) that have the potential for pharmacological action [22]. To improve the therapeutic effects of probiotics, next-generation probiotics (NGPs) are designed to be used in the treatment of many diseases using genetic engineering. Synthetic biology tools are being adapted to optimize and design the therapeutic efficiency of probiotics [23].

The expression pharmabiotic was first defined by Hill [24]. The term probiotics falls under the broader category of pharmabiotics, which are defined as “bacterial cells of human origin, or their products, that have a confirmed pharmacological impact on health or disease” [24]. Pharmabiotics encompass not only live organisms but also deceased organisms, their components, and bioproducts. Thus, pharmabiotics refer to bioactive agents, including probiotics, prebiotics, synbiotics, paraprobiotics, postbiotics, metabiotics, and NGPs, as well as living or non-living microorganisms, microbiological products, or nutrients with the potential for pharmacological action relevant to probiotics [25]. Pharmabiotics denote pharmaceutical probiotics that show clear evidence of promoting health benefits and eliciting positive physiological effects, or those with the ability to play a pharmacological role in treating diseases. As a result, pharmabiotics must meet two criteria: (i) they must provide physiological and pharmacological health benefits against diseases, and (ii) they must serve as both preventative and therapeutic approaches for medical conditions [26].

In summary, alternative treatment research remains important due to the frequency of side effects of the traditional drugs used today and their inadequacy in radical treatment. Among today’s alternative treatment research, which aims for more effective treatment and fewer side effects, interest in the treatment approach based on microbiota is increasing day by day. While several studies have independently explored the roles of probiotics, prebiotics, and synbiotics in relation to microbiota-associated diseases, most of these works address these components separately and do not provide an integrated evaluation of their pharmabiotic potential. To the best of our knowledge, a comprehensive review that collectively assesses the pharmacological roles of these biotic agents under the concept of “pharmabiotics” remains lacking in the current literature. In this review, the results and current status of scientific studies on pharmabiotics (probiotics, prebiotics, synbiotics, postbiotics, paraprobiotics, metabiotics, and NGPs) that affect the microbiota in favor of beneficial microorganisms are discussed.

## Probiotics

The gastrointestinal tract and gut microbiota of the human body are home to a diverse array of microorganisms [27]. There are more than 1000 bacterial species in the gut microbiota, 90% of which are *Firmicutes* and *Bacteroidetes* [28]. These microorganisms are crucial for human health due to their immunomodulatory properties and metabolic by-products, including short-chain fatty acids (SCFAs), vitamins, and compounds with analgesic, anti-inflammatory, and antioxidant properties, which they can influence metabolic and immunologic processes when they enter the bloodstream [29, 30]. Furthermore, alterations in the composition of intestinal microbiota can lead to complex diseases such as neurodegenerative disorders, obesity, asthma, diabetes mellitus, and inflammatory bowel disease. Therefore, maintaining a healthy gut microbiota is essential for promoting overall health [23].

**Fig. 2:**
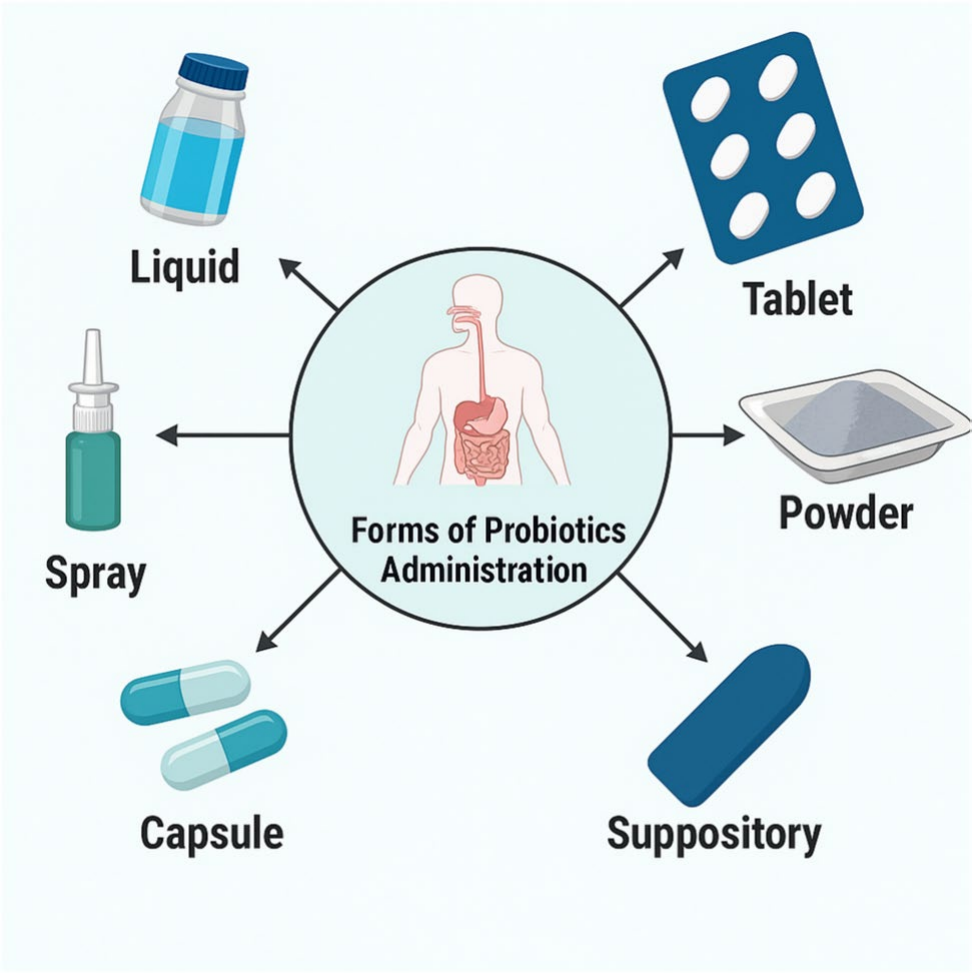
Forms of probiotics administration (generated using biorender.com)

Certain bacterial species can benefit their host through a relationship and are generally referred to as probiotic microorganisms. Probiotics have been defined as “live microorganisms that, when administered in adequate amounts, confer a health benefit on the host” by the International Scientific Association for Probiotics and Prebiotics (ISAPP) in 2014 [31]. Probiotics can be taken with various fermented dairy, non-dairy foods, or as supplements in the form of tablets, capsules, etc. (Fig. 2). The global probiotic market is expected to grow at an annual rate of 7.5% until 2030 [20]. Predominantly, they are classified within the genera *Lactobacillus* and *Bifidobacterium*; although, other bacteria such as *Bacillus* and *Escherichia coli*, as well as the yeast *Saccharomyces*, are also represented among them [5]. Probiotics should possess some qualities that facilitate their appropriate application. These qualities include tolerating bile, adhering to mucosal or epithelial cells, resisting antimicrobials, possessing bile salt hydrolase activity, stimulating the immune system, exhibiting antagonistic effects against pathogens, and demonstrating antimutagenic and anticarcinogenic properties [31, 32]. Properties of probiotics, such as resistance to acid and bile salts and adhesion, vary among different genera and species [33].

Probiotics play a vital role in maintaining gut health and promoting overall well-being in the host. They increase resistance to infectious illness, modify the host’s immune system, prevent the growth of tumour cells, protect against allergies, balance blood cholesterol, reduce the risk of long-term diseases, and lactose intolerance [34]. The health benefits of consuming probiotics have been documented in numerous randomized clinical trials [35]. A recent study by Talgatbekova et al. [36] is a specific example of a successful clinical application of probiotics. In this two-stage retrospective, prospective randomized controlled study conducted at NpJSC Astana Medical University, the nature and characteristics of intestinal dysbiosis in patients with hyperosmolar diarrhea with diseases of the pancreatic and biliary systems in a clinical setting were studied, and the effectiveness of probiotics as part of the treatment regimen was analyzed. In the first phase, a clinical assessment of the gut microbiocenosis of the patients involved was performed, and a total of 284 medical records were analyzed. In the second phase, the efficacy of the probiotics Biovestin-Lacto (containing *Bifidobacterium bifidum*, *Bifidobacterium adolescentis*, and *Lactobacillus plantarum* strains) and Normobact Forte (containing *Lactobacillus rhamnosus* strain) was maintained in a comparative, prospective randomized controlled trial. According to the results of the analysis, probiotics reduced the growth of opportunistic bacteria and yeast-like fungi while stimulating the indigenous natural microflora in the respective patients [36]. However, monitoring and determining the specific health benefits of administered probiotics has been challenging, as it requires understanding the unique metabolic functions of the administered probiotic as well as distinguishing it from native microbes already present in the host that could potentially produce similar outcomes [37]. Several theories have been suggested to clarify how probiotics exert their beneficial effects on health. These include improving the barrier functions of the intestinal lining [38], regulating the immune system [39, 40], adjusting the balance of gut bacteria [41], influencing overall metabolic responses in the body [42], and communicating with the central nervous system of the host [43, 44].

**Fig. 3:**
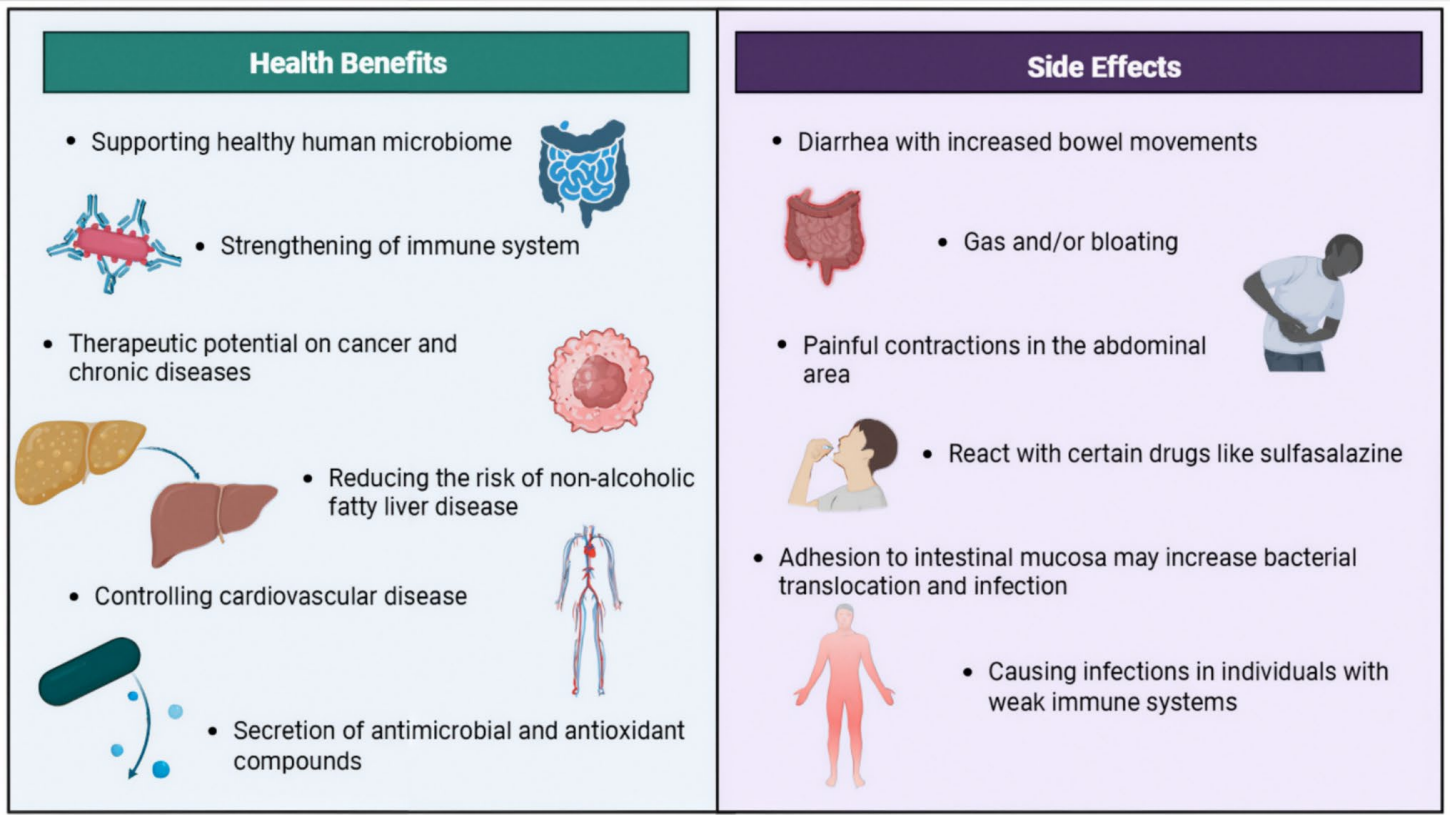
Some health benefits and side effects of probiotics (generated using biorender.com)

Probiotics benefit the host through multiple mechanisms, including immunomodulation and antimicrobial substance production. They also contribute to gastrointestinal tract functions, including electrolyte absorption and intestinal motility [45, 46]. However, some disadvantages limit the use of probiotics, such as their requirement for special storage conditions, such as temperature and humidity; having a short shelf life and a risk of antimicrobial resistance; and inability to be used in immunodeficiency [47]. While probiotics are generally considered safe for the majority of the population, their inappropriate or excessive use may pose potential health risks, particularly in individuals with underlying health conditions. Overuse of probiotics can disrupt the natural microbial equilibrium in the gut, potentially leading to small intestinal bacterial overgrowth (SIBO), gastrointestinal discomfort, bloating, or metabolic disturbances [48]. In some cases, prolonged administration of high doses of probiotics may interfere with the host’s immune signaling or lead to the colonization of undesired strains, which could cause unpredictable shifts in microbiota composition and function. Moreover, the use of multi-strain formulations without clear evidence of synergistic efficacy may increase the risk of antagonistic microbial interactions or gene transfer events, including those involving antimicrobial resistance genes [49]. Safety concerns are further heightened by the lack of standardized dosing regimens, strain-specific guidelines, and robust long-term safety data. These factors underscore the need for evidence-based recommendations and clinical oversight, especially when probiotics are used beyond general wellness purposes. Figure 3 indicates the health benefits and side effects of probiotics.

In normal health, *Bifidobacterium* and *Lactobacillus* are probiotics that predominate in the gastrointestinal microbiota. They are found in many products with health benefits [50]. *Bacteroides* and *Clostridium* have also been reported to have potential for the future, despite some species posing safety risks [51]. Furthermore, the yeast species *Saccharomyces* offers health advantages, especially in fermented milk products reported [52].

Probiotics produce antimicrobial compounds for self-defense. For example, they secrete lactic acid to lower pH, as in the case of *Lactobacillus* species [53, 54]. Probiotics have the ability to exert immunomodulatory effects in the host. They play a role in balancing pro- and anti-inflammatory cytokines. It has been reported that *Lactobacillus* strains limit the increase of human T cells [55]. Additionally, probiotics strengthen epithelial barrier integrity against mucosal ruptures and damage caused by *E. coli* by inducing the production of mucus granules from goblet cells that prevent pathogen penetration [54, 56]. It has also been noticed that probiotics produce a synergistic effect with native microflora in preventing enteropathogens [57].

It has been reported that most probiotics lose their viability after passing through the human digestive tract, but probiotics can still exert beneficial effects after digestion due to the health-promoting effects of dead probiotics and probiotic fragments [58, 59]. To maintain therapeutic efficacy, probiotics should be formulated as 10⁸–10⁹ CFU/g and should remain viable in the colon at 10⁶–10⁷ CFU/g. However, the low pH (pH 2) in the stomach environment reduces the viability of probiotics, so that the viability of probiotic bacteria reaching the colon remains below 10⁶–10⁷ CFU/g. Encapsulation of cells is an effective method to overcome this difficulty [60] (Table 1). Microencapsulation is a technology for packaging substances into miniature capsules that provide controlled release. In this process, core materials such as probiotics are coated with a protective wall material. Microencapsulation protects probiotics from adverse conditions such as processing, temperature, transportation, and intestinal transit. Encapsulation is achieved by spray drying, freeze drying, and similar techniques (Table 1) [61]. Spray-drying and freeze-drying techniques are the most commonly used techniques in commercial applications. In this way, probiotics are maintained viable throughout industrial processing and the digestive tract [62].

**Table 1:** Advantages and disadvantages of encapsulation methods

Encapsulation methods	Advantages	Disadvantages	References
Spray-drying	• Provides short processing time and lowest cost, high performance and easy applicability • Offers high efficiency with a single unitary process • Easy to store; creates capsules smaller than 100 µm • Easily combined with variety of options for coating materials • Provides rapid cell release by rapid dissolution of materials	• Drying conditions must be carefully monitored • High temperatures, dehydration, thermal and osmotic stress can cause losses • Damage to the surface structure of cells can trigger premature release of probiotics • High temperatures in the process lead to the death of many strains	[61–64]
Emulsion	• Easy to scale, a wide range of particle size and shape • Suitable lipid-based systems for protection against acids and oxygen • Applicable on industrial scale • Particles smaller than 300 µm can be formed • Provides high efficiency	• Emulsifiers may adversely affect cell viability • Significant loss of liquid phase • Liquid core may not be suitable for long-term stability • Formation of residues on the surface of particles	[61–64]
Extrusion	• Low cost for small scale applications • Offers precise working conditions • Wide range of coating materials • Easy and simple preparation process	• Scaling process is challenging due to slow particle formation • Large particles of 2–5 mm in size can form • Lower viability and encapsulation efficiency during storage	[61–64]
Coacervation	• Provides gentle and sensitive preparation conditions • High encapsulation efficiency • Allows encapsulation of large numbers of probiotic cells	• Costly and complex method • Coacervated probiotics need to be dried • Various dehydration techniques need to be applied	[61–64]
Freeze-drying	• Provides high durability • Provides higher viability than spray drying	• Long processing time and high cost • Ice crystals formed during freezing can damage the cell wall	[61–64]

**Fig. 4:**
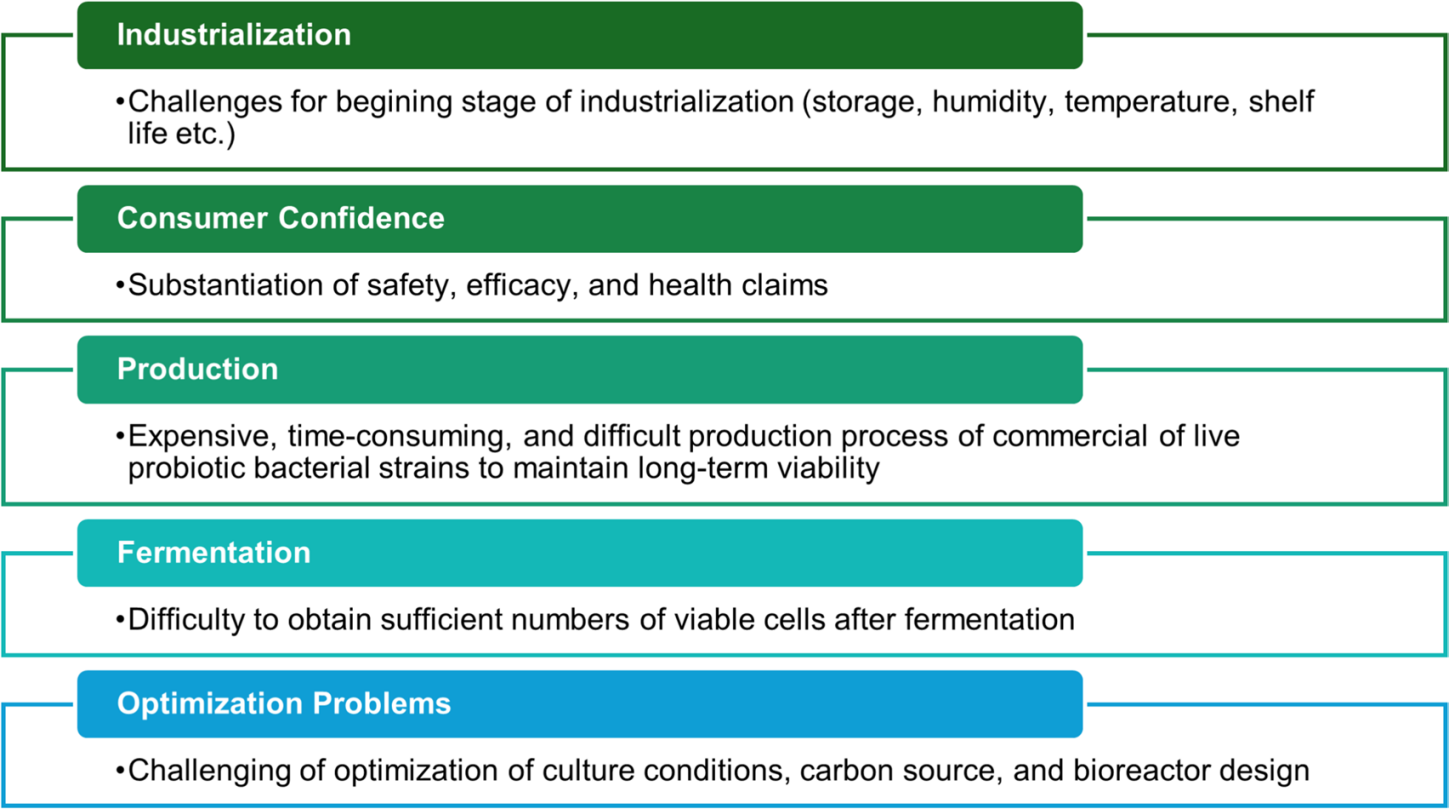
Challenges in probiotics technology

The development of PCR techniques in the 1980 s facilitated the identification of probiotics and enabled the detection of unculturable cells. However, the industrialization of probiotics is still in its infancy, and many challenges remain to be solved (Fig. 4) [65]. New fermentation strategies, e.g., immobilized cell technologies, membrane bioreactors, and continuous fermentation, hold promise for solving the challenges by supporting the proliferation of probiotic strains while maintaining pH and O₂ levels [66].

## Prebiotics

Nowadays, the concept of prebiotics is accepted as “a non-digestible food component that improves host health by selectively stimulating the growth and/or activity of certain bacteria in the colon that are beneficial to the host” [14]. They are dietary ingredients that undergo selective fermentation, leading to changes in the composition or function of the gut microbiota, thereby promoting host health. Unlike probiotics, which are live organisms, prebiotics consist of food components [67]. A range of natural sources provide prebiotics, including beans, legumes, starchy fruits, cereals, and soybeans [68]. Some prebiotics are carbohydrates/polysaccharides of plant origin (such as fructooligosaccharides, galactooligosaccharides, manno-oligosaccharides, and xylooligosaccharides, usually with a degree of polymerization between 4 and 30). However, some other compounds such as human milk oligosaccharides, phenols, and other phytochemicals, conjugated linoleic acid, and polyunsaturated fatty acids (PUFA) are also considered as a the class of prebiotic compounds [69]. Additionally, substances like galacto-oligosaccharides, inulin-type fructans, arabinoxylan, arabinoxylan oligosaccharides, chitin-glucans from fungi, and certain phenolic compounds are suggested to act as prebiotics due to their ability to enhance gut microbiota composition and promote positive health outcomes [15]. Dietary prebiotics must resist degradation by host enzymes. Furthermore, they must be selectively utilized and have sufficient evidence of health benefits for the target host [70].

Recently, the importance of plant prebiotic sources and plant prebiotic carbohydrates for healthy living based on a balanced microbiota (biosis) has been increasingly recognized. The main plant sources of prebiotics include vegetables (garlic, onions, celery, leeks, etc.), fruits (bananas, grapes, etc.), whole grain products, almonds, walnuts, hazelnuts, and legumes. The most well-known plant-derived prebiotic carbohydrates are fructooligosaccharides, inulin, and galactooligosaccharides. The raffinose family of oligosaccharides and resistant starch (the type that is not absorbed in the gastrointestinal tract) are also known as prebiotic carbohydrates. They are not absorbed from the gastrointestinal tract and support the growth of beneficial bacteria in the gut [71, 72]. Some polysaccharides found in plant cell walls such as xylans and pectins are also recognized as potential sources for prebiotic production [73]. There are also emerging prebiotic oligosaccharides (like raffinose, cellobiose, etc.) [74, 75]

## Synbiotics

**Fig. 5:**
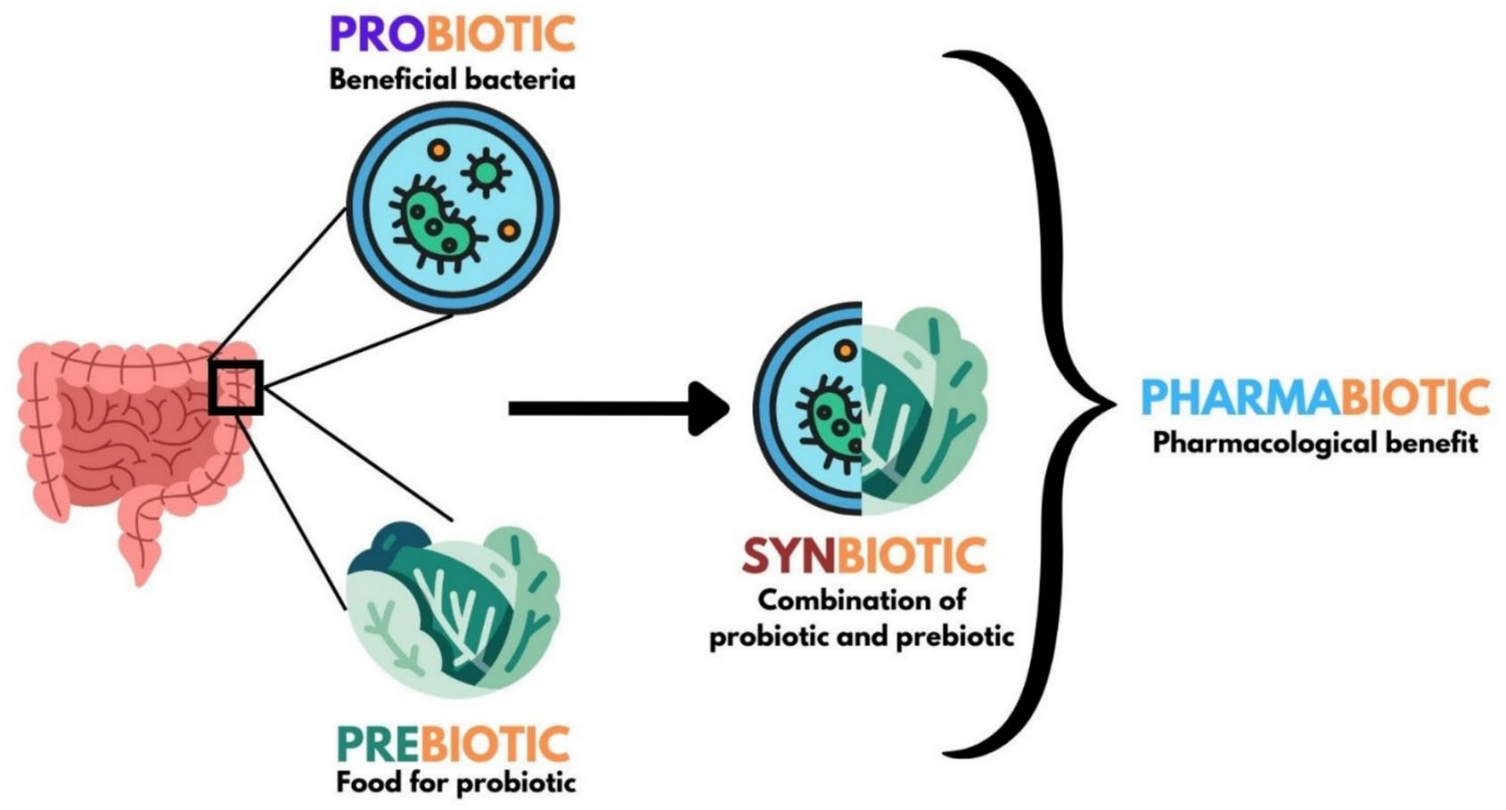
Schematic representation of terms related to synbiotics (generated using biorender.com)

Probiotics play a beneficial role in maintaining intestinal balance and act as a protective barrier for the digestive tract (Fig. 5). Conversely, prebiotics provide energy and nutrients for probiotic bacteria. Various probiotic strains rely on distinct food sources for sustenance, with each strain having a specific requirement. When a particular food source is available, only one strain can utilize it effectively, while the other strains may decline in number. Therefore, combining both components in a single product is expected to yield superior effects compared to using either probiotics or prebiotics alone [15]. From those combinations, the term synbiotic is defined as “a mixture of probiotics and prebiotics that beneficially affects the host by improving the survival and implantation of live microbial dietary supplements in the gastrointestinal tract (GIT), by selectively stimulating the growth and/or activating the metabolism of one or a limited number of health-promoting bacteria, and thus improving host welfare” by Gibson and Roberfroid in 1995 [76]. Prebiotics are primarily used as a specialized means to facilitate the growth and maturation of a probiotic strain within the intestinal tract. By using prebiotics, probiotic microorganisms become more resilient to environmental factors like oxygen levels, pH, and temperature in the digestive tract of the organism. Hence, synbiotics improve the survival of probiotic microorganisms in the GIT and help restore a balanced microflora in the GIT, offering a more significant impact on gut health compared to using probiotics or prebiotics alone [77, 78]. With combined probiotic and prebiotic properties, synbiotics are formulated to tackle potential obstacles to probiotic survival in the GIT [79]. Abdelshafy et al. [80] indicated that the application of oats as prebiotics to probiotics promoted the growth and survival of probiotics, and the probiotic-fermented oat synbiotic combination showed many potential biological activities such as antioxidant, antidiabetic, and antimicrobial activities higher than the activities of its components alone. It was also mentioned that this synbiotic combination could be used for many food applications such as the production of synbiotic beverages, synbiotic microcapsules, oat-based non-dairy yogurt, and gluten-free fermented foods [80].

Synbiotic promotes the growth of beneficial microbiota, balances metabolic activity in the gastrointestinal tract, supports the integrity of the intestinal structure, and prevents the proliferation of potential pathogens in the gut. Prebiotics, when fermented by probiotics and gut microbiota, result in elevated levels of SCFAs like acetate, propionate, and butyrate [17]. Among these, butyrate serves as a primary energy source for intestinal epithelial cells. SCFAs exhibit potential anti-inflammatory and antioxidant properties, influencing processes such as cell proliferation, differentiation, mucus secretion, and the maintenance of barrier function in the intestine [81].

## Paraprobiotics and Postbiotics

Paraprobiotics refer to the use of inactive microbial cells or cell fractions, while postbiotics refer to non-living bacterial soluble products or metabolic by-products secreted by probiotics that are beneficial to the host [82]. Mostly, peptidoglycans, surface proteins, and cell wall polysaccharides are classified as paraprobiotics, whereas molecules like secreted proteins, peptides, bacteriocins, and organic acids are categorized as postbiotics [83].

### Paraprobiotics

Paraprobiotics are a recently introduced concept to represent inactivated microbes known as “non-living, dead, inactivated, or ghost probiotics” that provide various health benefits to the host, including immunomodulatory, anti-inflammatory, antioxidant, and antihypertensive effects [20]. In a study on paraprobiotics, the immune defense mechanisms of paraprobiotics against viral infections were described based on clinical trial data on paraprobiotics until 2018 [84]. Meanwhile, the term “Para-psychobiotic” was introduced in a study showing the effects of *Lactobacillus* in reducing symptoms of extreme stress and improving sleep quality [85]. Paraprobiotics, whose benefits have been known since the 2000 s, have been used in the treatment of many diseases. Paraprobiotics can also be defined as non-living microbial cells, microbial fractions, cellular components, and bacterial lysates that improve the host health through interactions with the gut microbiome. These are usually composed of peptidoglycans, polysaccharides from microbial cell walls, cell surface proteins, and teichoic acid [86, 87]. In recent years, it continues to be extended with studies on its preclinical and clinical therapeutic properties [88]. For example, regular intake of *Lactobacillus* paraprobiotics has been shown to significantly affect intestinal functionality due to brain-gut interaction [89]. In another study conducted by Murata et al. on paraprobiotic *Lactobacillus* supplementation, it was reported that the supplement reduced cold symptoms and mental function problems [90]. Preclinical and clinical studies have also shown that paraprobiotics can be used safely in preterm infants [91, 92].

### Postbiotics

Recently, interest in the use of alternative biotherapeutic products has increased. It focuses on the use of non-living bacteria and microorganism-derived cell components, as well as containing metabolites that may have advantages over probiotics and be safer to use in vulnerable populations. Postbiotics has been defined as “preparation of inanimate microorganisms and/or their components that confers a health benefit on the host” [93]. The rise of the postbiotics term is based on the extensive field of research focusing on the microbiota and its impact on both health and disease. Postbiotics are usually heterogeneous mixtures of cellular structures. These are mostly metabolites such as teichoic acids, exopolysaccharides, and peptidoglycan bacteriocins. These substances can occur as fermentation products or can be obtained by the breakdown of bacteria [18]. Three main mechanisms are involved in the efficacy of postbiotics: strengthening of the epithelial barrier, reduction of immune responses, and modulation against pathogens. The main potential therapeutic advantages of postbiotics are to sustain steady under normal environmental conditions and the absence of viability prerequisites. Postbiotics have been reported to have direct antimicrobial properties [94]. For example, organic acids belonging to probiotics such as lactic acid bacteria and bifidobacteria have been shown to have a dose-dependent antimicrobial effect, primarily against Gram-negative pathogens [95, 96].

Compared to the probiotics, postbiotics carry less risk of antimicrobial resistance. Antibiotics have broad-spectrum bactericidal properties. While they kill pathogenic bacteria, they also kill probiotics, thus causing symptoms such as diarrhea and indigestion. However, postbiotics do not cause such problems and can be used in conjunction with antibiotics. Thus, they can quickly regulate the intestinal microbiota during and after antibiotic treatment. They are more stable and safe and easier to store at room temperature that is suitable for transportation. Thanks to their functional properties, postbiotics provide better stability, texture, and taste than probiotics, positively impact the physicochemical and sensory properties of the final product, and can therefore be used as functional adjuvants [97]. Postbiotics are currently used in the fermented food industry, and they are seen as an effective complementary treatment modality and a driving force for the development of a comprehensive healthcare industry [98]. Furthermore, these benefits provide significant advantages in treatment approaches and effects on human health despite some of the limitations associated with the use of probiotics [99, 100]. Seong et al. [101] investigated the effectiveness of heat-killed *Lactobacillus casei* DKGF7 on symptomatic relief of irritable bowel syndrome (IBS) in a rat disease model and achieved lower serum corticosterone levels, lower colonic inflammatory cytokine levels, and higher tight junction proteins expression. Thus, they showed that postbiotics may be a potential therapeutic strategy for the treatment of IBS [101]. In another study, De Marco et al. [102] investigated the anti-inflammatory activity of probiotic metabolites which are cell-free supernatants of *Lactobacillus acidophilus*, *Lactobacillus casei*, *Lactococcus lactis*, *Lactobacillus reuteri*, and *Saccharomyces boulardii*. They found that the metabolites produced by these probiotics downregulated the expression of PGE-2 and IL-8 in human colon epithelial HT-29 cells and also significantly showed dose-dependent radical scavenging activity. Thus, probiotic metabolites can be used as adjuvants in anti-inflammatory treatment to prevent and reduce intestinal inflammation [102].

## Metabiotics

Recent studies have reported that probiotics can create low molecular weight (LMW) bioactive molecules that can be used as drugs, bioactive food additives, or as enriching microbial components of functional foods to replenish the gut microbiota and improve health by digesting food components and compounds released by host cells [103]. These bioactive molecules are called metabiotics. Metabiotics are defined as metabolic by-products secreted by living bacteria during fermentation or after bacterial lysate and provide physiological benefits to the host [22]. The term metabiotics, which is obtained from the Greek prefix meta- containing the meaning of changing, was first defined by Shenderov in 2013. As the name suggests, metabiotics have the ability to initiate numerous hormonal and neurochemical processes [103]. Metabiotics include components of probiotic microorganisms, their metabolic by-products, and signaling molecules with known chemical structures that have the potential for pharmacological action. These components not only have the ability to improve specific physiological functions customized specifically to the host, but they also display regulatory, metabolic, and behavioral properties. Principal elements of metabiotics are SCFAs, PUFAs, bacteriocins, polysaccharides, and peptides [21]. Metabiotics have numerous health outcomes on the host with their wide range of functional compounds and the ability to optimize host-specific physiological functions. It is also known to have positive effects on the host, such as decreasing oxidative stress, balancing blood pressure, providing immune modulation, anti-inflammatory, and anticancer effects [22]. In a clinical study, probiotic *Lactobacillus rhamnosus* was shown to produce metabiotics with antigenotoxic and cytotoxic properties against colon cancer [104]. Metabiotics possess favorable characteristics such as stability, precise dosing, and safety, which make them suitable candidates for extending the shelf life of food products [105]. Their effectiveness in food preservation is primarily attributed to several well-documented mechanisms. One of the key mechanisms is their antimicrobial activity, resulting from the production of organic acids (e.g., lactic acid, acetic acid), bacteriocins, hydrogen peroxide, and various enzymes that inhibit the growth of spoilage and pathogenic microorganisms [86]. Bacteriocins such as nisin and pediocin, commonly synthesized by lactic acid bacteria, exert bactericidal effects by disrupting microbial cell membranes and interfering with vital cellular functions [106]. In addition, metabiotics exhibit antioxidant properties, which help prevent oxidative spoilage by scavenging free radicals and inhibiting lipid peroxidation, thereby maintaining the sensory and nutritional quality of food [86]. The acidification of the food matrix through organic acid production further suppresses microbial growth by reducing pH and creating inhospitable conditions for spoilage organisms [107]. Collectively, these multifunctional properties underscore the potential of metabiotics as safe and effective agents for food preservation and shelf-life extension.

## Next-Generation Probiotics (NGP)

Dynamics related to the human microbiota have been linked to many diseases such as inflammatory bowel disease, cancer, obesity, and autoimmune diseases. A recent emerging paradigm for the treatment of diseases with probiotics is the administration of live engineered organisms, also referred to as NGPs. Recently, engineered non-conventional probiotics are being tested as biotherapeutic agents in the treatment of various diseases, the design of which is limited by the availability of genetic tools. Next generations of probiotics are genetically engineered. Plasmids, DNA molecules, are used to genetically modify probiotics [108]. Here, some factors limit the use of plasmids, such as strain-to-strain variation, low plasmid copy number, and the presence of endonuclease activity inside cells [109]. Alternatively, the gene of interest can be inserted into the probiotic chromosome using genome engineering tools, which increases the genetic stability of the probiotic [110]. However, the efficacy of such therapies may be limited by the overall performance of the organism in the harsh and nutrient-limited environment of the gut [111].

NGPs have very important beneficial effects on host intestinal health and other health factors. The mechanisms of action of NGPs on host intestinal health and other intestinal health factors may vary depending on the strains and formulations [59]. SCFAs, lactic acid, and antimicrobial compounds produced and secreted by NGPs play an important role in regulating the intestinal microbiota and maintaining its balance, and strengthening the immune system by killing pathogenic microorganisms. NGPs can also regulate bile acid metabolism, which plays a role in various metabolic and inflammatory disorders. This regulatory ability of NGPs indicates that they have therapeutic potential on the host [112]. In addition, NGPs can support mucus production in the gut and strengthen the intestinal epithelial barrier. They compete with pathogenic microorganisms in the intestines, reducing their growth and preventing them from entering the body [113]. Recent studies have defined strains such as *Prevotella copri*, *Christensenella minuta*, *Parabacteroides goldsteinii*, *Akkermansia muciniphila*, *Bacteroides thetaiotaomicron*, *Faecalibacterium prausnitzii*, and *Bacteroides fragilis* as potential NGPs that offer considerable promise in the prevention and treatment of dysbiosis-related diseases [114]. Miranda et al. demonstrated that the NGP potential of live and inactivated *Akkermansia muciniphila*, when administered orally, has a systemic immunomodulatory effect that could be used in the treatment of food allergy [115]. Lee et al. showed that recombinant *Lactococcus lactis* expressing LZ8 protein appears to be a promising low-cost drug for improving nonalcoholic fatty liver disease by exhibiting anti-inflammatory effects [116].

To increase the therapeutic utility of engineered probiotics, optimization studies are also being conducted [117, 118]. It has been reported that engineered probiotics can also be used as live diagnostic tools to sense and secrete therapeutic protein in response to environmental triggers [119, 120]. Recent studies in this field show that probiotics engineered with therapeutic enzymes have great potential in the treatment of metabolic diseases such as phenylketonuria, enteric hyperoxaluria, homocystinuria, hyperammonemia, and diabetes [121, 122]. Successful therapeutic administration of this next generation of probiotics is challenging for many reasons, including reduced viability in the complex intestinal environment. Colonization resistance exerted by commensal gut microbiota through competition for nutrients may also be a major barrier to their growth and survival [123]. The shortcomings of current studies on NGPs are that they are not specific to a single disease and the molecular mechanisms underlying their therapeutic effects have not been sufficiently elucidated [23].

## Fecal Microbiota Transplantation (FMT)

**Fig. 6:**
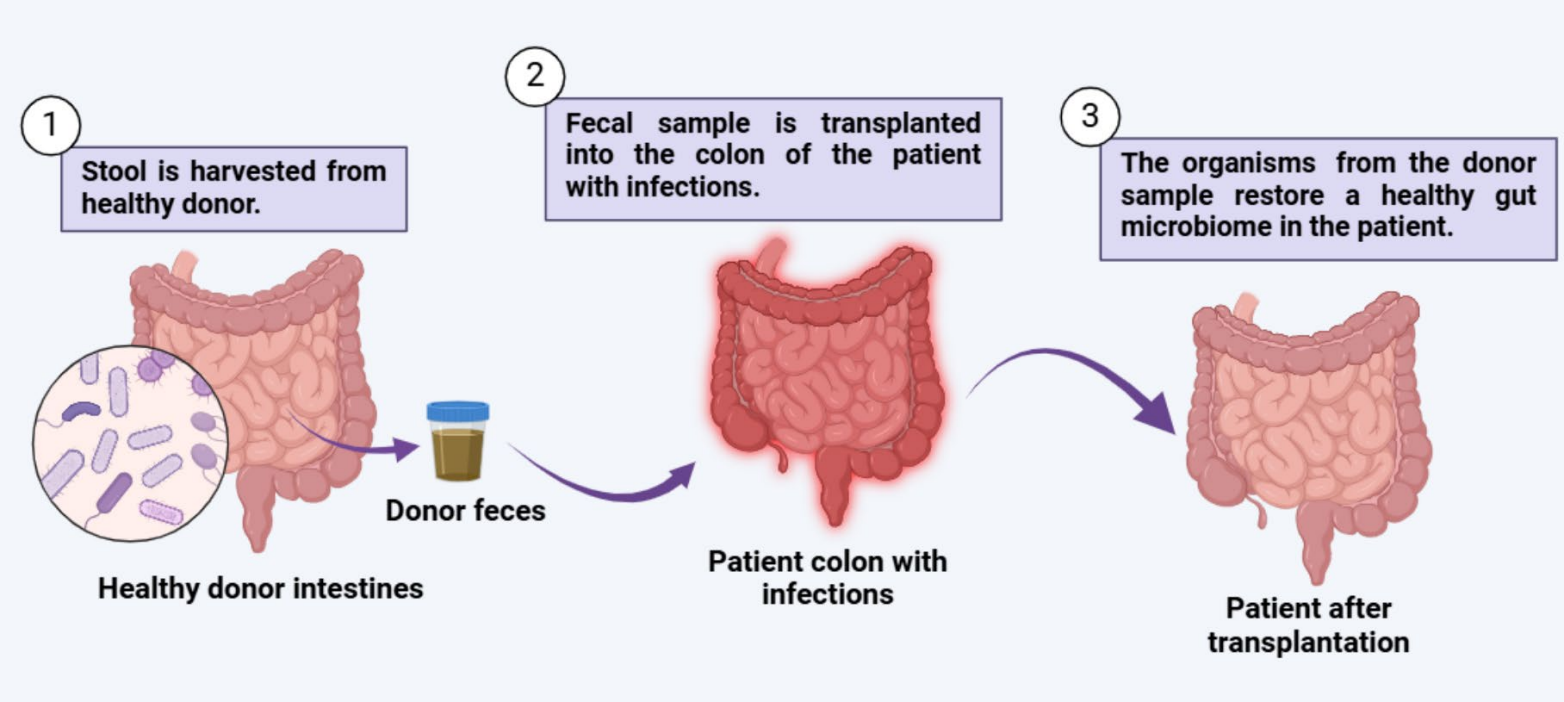
Schematic representation of fecal microbiota transplantation process (generated using biorender.com)

**Fig. 7:**
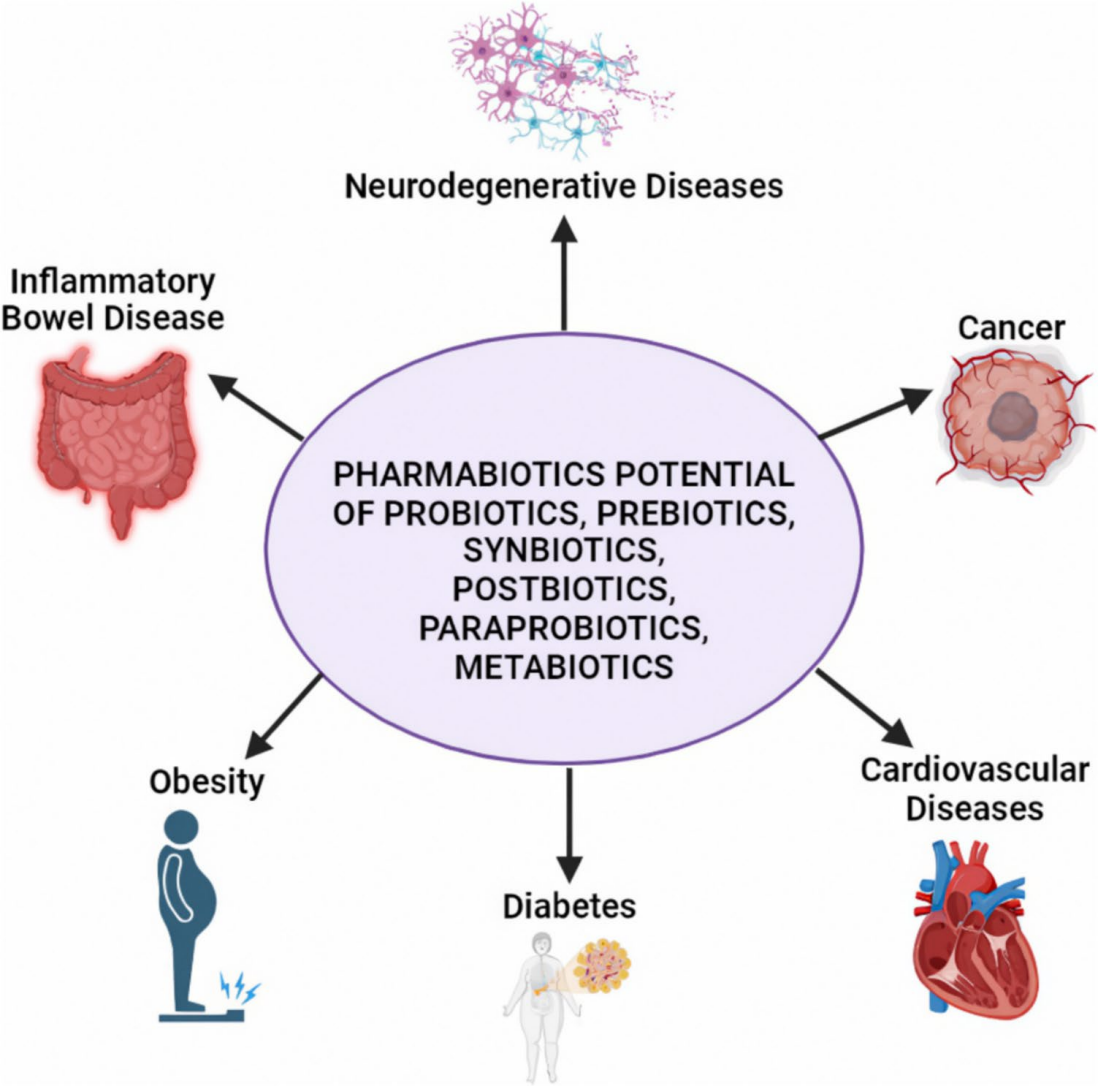
Schematic representation of pharmabiotic potential of probiotics (generated using biorender.com)

Fecal microbiota transplantation (FMT), also known in the medical literature as fecal bacteriotherapy, stool transplantation, or fecal transfusion, is the process of filtering and diluting stool from a healthy human donor and introducing it into the gastrointestinal tract of the recipient via colonoscopy, enema, nasogastric tube, or in capsule form. FMT is a therapeutic modality that directly alters the intestinal microbiota of the recipient, with the aim of normalizing the host microflora and thus treating the disease(s) caused by the imbalanced microflora (Fig. 6). FMT has a long history and was first used orally by Ge Hong in China in the fourth century as “Yellow Soup” to treat food poisoning and severe diarrhea [124]. In modern medicine, FMT was first used by Eiseman et al. in 1958. Since 2013, when the Food and Drug Administration (FDA) approved FMT, this treatment has become widespread. Today, FMT applications are widely used not only to treat gastrointestinal disorders (Fig. 7). but also non-gastrointestinal diseases.

Although current evidence suggests that FMT is a generally safe treatment modality with few side effects, the long-term consequences of FMT have not been fully elucidated. Therefore, the long-term efficacy and side effects of FMT need to be monitored [125]. Donor selection should be performed prior to FMT with questionnaires, interviews, blood tests, and fecal examinations to reduce and prevent any side effects of FMT. Recently, personalized approaches to microbiome modulation by FMT have been proposed [126]. The reason is the gut microbial variation between human populations, and this variation can cause difficulty in FMT. As variations in the microbiome exist among human populations, FMT, probiotic, and prebiotic applications should allow for more targeted and personalized recommendations based on diet, ancestry, geography, and physiology, as well as microbial phylogenetic, metagenomic, and metabolic considerations [126].

Today, it is known that the gut microbiota is of critical importance in human health and disease prevention. Disruption of the natural balance in the gut microbiota (dysbiosis) is associated with chronic diseases. Currently, dysbiosis in the gut microbiota can be modulated by diet, pharmabiotics including probiotics, prebiotics, postbiotics, and fecal microbiota transplantation. An effective strategy for gut microbiota modulation for the treatment of chronic diseases is needed. This approach is personalization and targeted modulation. Analysis of individual gut microbiome, metabolome, strain specificity, and clinical data can reveal specific changes in the diversity, composition, and function of the gut microbiota, and these analyses enable personalized and targeted gut microbiota modulation through the administration of beneficial microorganisms, their consortia, metabolites, and effective combinations [127].

FMT has proven effective for the treatment of *Clostridioides difficile* infections, which are frequently caused by antibiotic usage. Beyond this application, it holds potential for the addressing of numerous additional disorders, including colorectal cancer, ulcerative colitis, Crohn’s disease, metabolic syndrome, obesity, and autism. While functionally similar to other organ transplants, FMT is not rejected by the body, provides a potentially strategy, and integrates seamlessly with the recipient's microbiota [128]. The essential conditions for FMT are a healthy donor with an appropriate microbiome; the correct selection of a suitable recipient; the exclusion of pathogens such as bacteria, viruses, and infections from the recipient; and the maintenance of host homeostasis [129].

Unlike traditional therapies for IBD, FMT alters the microbial environment, affecting the host’s immune system, offering a safer alternative treatment. Recent studies have shown that FMT has indicated positive effects in combination with traditional endoscopic and clinical treatments in preventing exacerbations and complications of ulcerative colitis (UC) [130]. Paramsothy et al. analyzed colon and fecal samples collected before and after a randomized controlled trial of FMT in patients with active UC, showing that FMT increased microbial diversity and altered its composition. They indicated that the diversity included *Eubacterium*, *Roseburia*, short-chain fatty acids, and secondary bile acid biosynthesis in patients who experienced remission, while it included *Fusobacterium*, *Sutterella*, and *Escherichia* species in patients who did not experience remission [131].

Although studies have shown that FMT is an alternative treatment method for UC patients, the results of studies are not sufficient to use FMT in the treatment of UC in clinical practice. In addition, evidences have indicated that the effect of FMT is transient, as disease relapses occur after short-term positive results in FMT treatment. Therefore, further research is required to achieve long-term efficacy and to use it as maintenance therapy in UC [132].

Case reports and pilot studies supported the administration of FMT in the treatment of celiac disease (CD); although, there is a lack of evidence for the use of FMT as a potential treatment in clinical practice [128]. Sokol et al. [133] conducted a randomized, single-blind, sham-controlled pilot trial of FMT in adults with CD. This is the first randomized, sham-controlled pilot study to evaluate FMT in Crohn’s disease and found a nonsignificant reduction in the incidence of flares in the FMT group compared to the sham group. The low similarity between donor and recipient microbiota in some patients suggests that a single FMT may not be sufficient to cause significant changes in these patients. However, when clinically important values such as C-reactive protein levels (CRP) were evaluated, FMT was found to be more advantageous than sham transplantation for several clinically relevant endpoints. Larger studies are needed to confirm these results [133].

Since FMT regulates the health of the host microbiome and strengthens the intestinal barrier to maintain its function, when healthy microbiota is given to an organism whose microbiome health is impaired due to pathogenesis, this balance can be restored. For this reason, FMT has become a more applicable treatment method to restore microbial balance and functionality [134]. Transplanted gut microbes not only affect cancer antigens but also act on the immune system by stimulating innate and adaptive immune cells. In addition, the mechanisms of action of FMT include reducing the effectiveness of the host’s immune suppressors, modulating immune response to cancer cells, and modifying the tumor microenvironment [135]. Yu et al. investigated the effects of FMT on the progression of colorectal cancer (CRC). FMT was performed by transferring intestinal microbes from healthy mice to CRC mice via enema. FMT reversed the dysregulated intestinal microbiota of CRC mice, halted cancer progression, and increased the survival of CRC mice. In addition, FMT reduced the accumulation of immune suppressor cells while promoting the infiltration of immune cells that can directly kill cancer cells. They showed that FMT prevented the development of CRC by regulating intestinal microbial disorder and triggering immune cells [136]. Chen et al. combined submucosal injection, an orthotopic xenograft rectal cancer model generation method, with FMT, an effective method for delivering target bacteria and achieving sufficient colonization. Their results indicated that this combination is a promising approach for creating an animal model of CRC and treating it with FMT for future studies [137].

FMT has shown clinical success, particularly in treating recurrent *Clostridioides difficile* infections; however, its broader application faces several challenges and limitations. These include the lack of standardized donor screening and stool processing protocols, which raise concerns about safety, consistency, and reproducibility of the treatment [131, 138]. Potential risks involve transmission of pathogens, adverse immune responses, and unintended alterations to host metabolism or microbiota balance [139]. Additionally, regulatory uncertainty and ethical concerns regarding donor anonymity and long-term effects—such as increased susceptibility to metabolic disorders or immune-mediated diseases—limit its clinical use [140]. Despite its promise, FMT requires further research to optimize delivery methods, define appropriate indications, and ensure long-term safety.

## Mechanisms of Action of Pharmabiotics

Recently, probiotics have been shown to play a role in the therapeutic effects of various diseases through their capacity to influence various pathways in the host. Molecular mechanisms underlying the therapeutic effects in which probiotics play a role include competitive exclusion of pathogens for adhesion sites, improvement of the intestinal mucosal barrier, intestinal immunomodulation, and neurotransmitter synthesis. This mechanism of action may be mediated by probiotics related pharmabiotics. Studies show that probiotics, postbiotics, paraprobiotics, and metabiotics have effects on basic biological signaling pathways such as NFκB, MAPK, Akt/PI3 K, and PPARγ. In vitro, in vivo, and clinical studies on probiotics show that probiotics exert their blood lipid-lowering effects by activating the AMPK signaling pathway [141]. For example, Hasanian-Langroudi et al. reported that since dynamic interactions between diet and gut microbiota play an important role in the pathogenesis of T2DM, probiotics may help in the treatment of T2DM by improving the dysbiosis. In the related study, the authors reported that modification of gut microbiota with probiotic strains of *Lactobacillus* and *Bifidobacterium* improved glucose metabolism and insulin resistance. It has been suggested that the antidiabetic effects of these probiotics may be due to their effect on signaling pathways such as NF-κB, PI3 K/Akt, and Nrf2 [142]. In another study, Xiao-hang et al. [143] investigated the effect of probiotic treatment in Alzheimer’s disease and its potential molecular mechanisms through transcriptomic analysis and western blotting. In this study, they used 6-month senescence-accelerated mouse prone 8 (SAMP8) and senescence-accelerated mouse resistant 1 (SAMR1). SAMP8 mice were treated with probiotic-2 (P2, probiotic mixture of *Bifidobacterium lactis* and *Lactobacillus rhamnosus*) and probiotic-3 (P3, probiotic mixture of *Bifidobacterium lactis*, *Lactobacillus acidophilus*, and *Lactobacillus rhamnosus*). Transcriptomic analysis results showed that the chemokine signaling pathway was the most significantly enriched signaling pathway between SAMP8 and SAMR1 mice. By Western blot assay, a significant change in the phosphorylation level of downstream AKT/GSK-3β between SAMP8 and SAMR1 groups was demonstrated, which could be reversed by P2 and P3 treatment. The results of the study showed that multi-strain probiotic treatment could ameliorate cognitive disorders and pathological changes, including nerve damage, Aβ and Tau pathology, and neuroinflammation, in SAMP8 mice by regulating the phosphorylation of the AKT/GSK-3β pathway [143]. It has been reported that the therapeutic effects of probiotics occur through microbiota-mediated pharmacological mechanisms of action [8]. In the therapeutic effects of probiotics in lipid metabolism disease, it has been reported that probiotics regulate lipid synthesis by activating the AMPK signaling pathway and effectively improve lipid metabolism disease through the HMGCR/FXR/SHP signaling pathway [8].

## Pharmabiotics in Pharmacology

### Pharmabiotics in Gastrointestinal Diseases

Various gastrointestinal diseases such as IBD, including UC, Crohn’s disease (CD), and indeterminate colitis (IC) cause significant morbidity and mortality worldwide each year [11]. In addition, IBD, which includes UC and CD, causes symptoms such as diarrhea, abdominal pain, fever, iron deficiency anemia, and weight loss [144]. Individuals diagnosed with IBD often explore complementary and alternative medicines, driven by concerns over potential side effects or the perceived inadequacy of conventional drug therapies [145]. However, there is considerable patient interest in utilizing probiotics as a treatment option. Studies investigating the efficacy of probiotics in managing IBD have been ongoing since 1997 [11, 146].

Some authors propose that the impact of probiotics on initiating, sustaining, and prolonging periods of remission in UC is linked to the immunomodulatory function of specific microorganisms. Tamaki et al. [147] conducted a randomized, double-blinded, placebo-controlled trial to assess the effectiveness of *Bifidobacterium longum* 536 (BB536) supplementation in inducing remission in patients with active UC. Fifty-six patients with mild-to-moderate UC were randomly assigned to receive either BB536 or placebo for 8 weeks. Results showed that 63% of patients receiving BB536 achieved clinical remission at week 8 compared to 52% in the placebo group. The BB536 group also experienced a significant decrease in UC disease activity index (UCDAI) scores and in the Rachmilewitz endoscopic index (EI) and the Mayo subscore at week 8, whereas the placebo group did not show significant changes. One patient in the BB536 group reported a mild side effect, but no other adverse effects were observed [147]. In another study, Ebrahiminejad et al. investigated the probiotic effects of live and pasteurized forms of *Lactobacillus brevis* IBRC-M10790 on IBD in an in vitro co-culture model. In the model using Caco-2 intestinal epithelial cells and RAW264.7 macrophages, *L. brevis* was observed to decrease proinflammatory cytokines (IL-6, IL-1β, TNF-α) and increase anti-inflammatory cytokine IL-10. In addition, *L. brevis* increased tight junction proteins such as ZO-1, E-cadherin, and occludin, which strengthen the epithelial barrier. It was found that pasteurized *L. brevis* showed a higher protective effect than the live form. Thus, they showed that *L. brevis* has the potential to alleviate inflammation in IBD [148].

Apart from studying probiotic bacteria for gastrointestinal disease treatment, a significant attention has been given to exploring their immunomodulatory properties when administered in synbiotic combinations. Martino et al. [149] investigated the effect of a combination supplementation of beneficial probiotic strains (*Saccharomyces boulardii*, *Lactobacillus rhamnosus*, *Lactobacillus acidophilus*, and *Bifidobacterium breve*) and amylase in SAMP1/YitFc (SAMP) mice with CD-like ileitis. Histological results showed that ileitis severity was reduced in mice treated with probiotics and amylase. 16S ribosomal RNA and GC–MS analyses revealed an increase in the abundance of *Lachnoclostridium* and *Mucispirillum schaedleri* species in this group, and this increase was associated with the production of short-chain fatty acids. They indicated that the designed probiotic formulation, which alters intestinal genetic pathways and microbial regulations, showed beneficial effects in reducing the severity of CD-like ileitis in the SAMP mouse model [149]. Steed et al. tested the effects of a synbiotic containing *B. longum*, inulin, and oligofructose on Crohn’s disease patients via a double-blind trial. Patients were given either the synbiotic or a placebo, and their clinical status was evaluated alongside rectal biopsies taken. Results revealed significant improvements in clinical outcomes, with synbiotic consumers experiencing reduced disease activity and histological scores. Notably, TNF-α expression decreased significantly at 3 months, and synbiotics intake also boosted mucosal bifidobacteria levels [150].

S. Guo et al. conducted a randomized, double-blind, placebo-controlled study with 69 participants to evaluate the potential of postbiotics in alleviating symptoms of chronic diarrhea. Postbiotic administration resulted in significant improvements in stool consistency, frequency of bowel movements, urgency, and anxiety. It also increased beneficial intestinal bacteria such as *Dysosmobacter welbionis* and *Faecalibacterium prausnitzii* while decreasing potential pathogens such as *Megamonas funiformis*. Metabolomics analysis showed that beneficial metabolites (α-linolenic acid and p-methoxycinnamic acid) increased and diarrhea-associated metabolites decreased. In addition, fecal butyric acid levels increased, and aromatic amino acids such as phenylalanine and tryptophan and related metabolites decreased. They demonstrated that postbiotics were effective in alleviating diarrhea and that this effect was mediated by modulating tryptophan metabolism [151]. Postbiotics are a hopeful option for the prevention and treatment of gastrointestinal diseases by increasing intestinal barrier integrity and reducing inflammatory cytokine levels due to their stability and safety compared to probiotics [152].

### Pharmabiotics in Cancer

Numerous in vitro and in vivo animal studies and human clinical trials have demonstrated that probiotics exert pharmabiotic effects, playing a significant role in the regulation of cancer cell proliferation and apoptosis [153]. Probiotics exert their anticancer effects through several mechanisms, including the promotion of beneficial changes in intestinal flora, modulation of metabolic activity, binding and degradation of carcinogenic compounds, immunomodulation to alleviate chronic inflammation, reduction of intestinal pH, and inhibition of enzymes involved in the production of potential carcinogens [32]. Tiptiri-Kourpeti et al. studied investigating the effects of *Lactobacillus casei* ATCC 393 on colon cancer. The study found that administration of live *Lactobacillus casei* or its components inhibited the growth of colon cancer cells in vitro and in vivo. Furthermore, oral administration of live *Lactobacillus casei* significantly reduced tumor volume in mice, accompanied by upregulation of the TNF-related apoptosis-inducing ligand TRAIL and downregulation of survivin [154]. Jacouton et al. evaluated the protective effects of oral treatment with *Lactobacillus casei* BL23 against CRC. CRC was induced in mice, and *L. casei* BL23 treatment significantly reduced tumor development, as evidenced by lower histological scores and proliferative index values. Furthermore, *L. casei* BL23 exhibited immunomodulatory effects by downregulating IL-22 and antiproliferative effects by upregulating caspase-7, caspase-9, and Bik. Treatment with *L. casei* BL23 also tended to restore dysbiosis induced by CRC [155].

Prebiotics hold promise as adjuvants in cancer therapy, particularly for colon cancer. By providing readily available carbon sources for intestinal microbes, they stimulate the production of butyric acid and other substances that support the apoptosis of tumor cells. Li et al. focused on selecting prebiotics that enrich bacterial taxa promoting anti-tumor immunity. In experiments with C57BL/6 mice, adding prebiotics inulin or mucin to the diet induces anti-tumor immune responses and inhibits BRAF mutant melanoma growth in a mouse model. Inulin and mucin drive distinct changes in the microbiota, with inulin limiting tumor growth in colon cancer and NRAS mutant melanoma models and enhancing the efficacy of a MEK inhibitor against melanoma while delaying drug resistance [156].

Numerous studies have highlighted the significance of prebiotics, probiotics, and synbiotics in adjunctive tumor therapy, showing not only a favorable impact on tumor progression but also effective mitigation of postoperative infectious complications [157]. Khazaei et al. investigated the effect of synbiotic supplementation in reducing chemotherapy side effects in women undergoing breast cancer treatment. Sixty-seven patients were assigned to either the synbiotic or placebo group and received synbiotics twice daily for 8 weeks. The results showed a significant reduction in the severity of abnormal defecation and fatigue in the synbiotic group. Nausea/vomiting and anorexia were also reduced, but this reduction was not statistically significant compared to the placebo group. They stated that synbiotic supplementation could be considered as a potential adjuvant therapy to alleviate some common side effects during chemotherapy [158]. In another study, the synbiotic potential of salicylic acid and *Lactobacillus rhamnosus* GG (LGG) was investigated. Salicylic acid significantly increased the coaggregation of LGG with *E. coli*, increased its antioxidant properties, and also contributed to the cytotoxic effects of LGG against human colon cancer cells [159].

Postbiotics that exhibit anti-cancer potential inhibit the growth and proliferation of cancer cells, increase apoptotic effects, regulate immune responses, and suppress mutagenesis and carcinogens [160]. Nowak et al. studied the effect of post-fermentation medium (PFM) and cell extracts (CEs) of various LAB strains against colon (Caco-2) and cervix (HeLa) cancer cell lines. They showed that PFM and CEs exhibited a potent dose-dependent antiproliferative activity against Caco-2 cells and induced oxidative stress in Caco-2 cells. Moreover, PFM of *L. plantarum* 0991 and *L. brevis* 0983 induced apoptosis in Caco-2 cells via mitochondrial signaling [161]. In another study, the anti-proliferation effects of postbiotics against HT-29 cells, a colon cancer cell line, were investigated. Postbiotics produced by ultrasonication from two *Lactobacillus* strains (*Lactobacillus* sp. La1 and La2) were thought to provide important information in the development of anticancer research by reducing the viability of HT-29 cells [162]. Additionally, postbiotics play important roles in increasing the effectiveness of standard cancer treatments by modulating immune responses and enhancing their anti-cancer activity [163]. Lu et al. reported that oral administration of extracellular vesicles (EV) derived from *Lactobacillus rhamnosus* GG (LGG-EV) synergistically improved the efficacy of anti-PD-1 immunotherapy against colorectal cancer. LGG-EV regulated intestinal immunity by increasing the CD8 + T/CD4 + T cell ratio in mesenteric lymph nodes and enhancing the ratio of MHC II + DC cells, CD4 + T cells, and CD8 + T cells in tumor tissues. Significant changes were observed in the levels of serum metabolites associated with microbiota and antitumor effects [164].

### Pharmabiotics in Metabolic Diseases

Metabolic disorders, such as diabetes, obesity, and CVD, result from irregularities in the physiological processes that govern metabolism within the body [94]. Metabolic disorders can arise from various causes, including hereditary factors, habits and behaviors, dietary choices, and environmental factors. Furthermore, metabolic disorders are often associated with dysbiosis of the gut microbiota [165]. The concept of pharmabiotics stems from extensive research focused on the microbiota and its effects on metabolic disorders.

CVD are a class of metabolic disorders that encompass conditions affecting the heart or blood vessels, such as strokes and hypertensive heart diseases. WHO states that these diseases are the primary cause of illness and death globally [166]. Several important dietary factors can reduce the risk of cardiovascular diseases: (i) decreasing LDL cholesterol by limiting saturated fat intake; (ii) lowering triglyceride levels by cutting down on sugar and processed foods; (iii) reducing homocysteine levels through supplementation with vitamins B6, B12, and folic acid; and (iv) boosting antioxidant activity by consuming more fruits and vegetables [167]. However, altering one’s diet can also change the microbial and metabolic makeup of the intestines, which is linked to various physiological and pathological states, including cardiovascular diseases. The composition of gut microbiota has been associated with chronic heart conditions. Understanding these interactions is crucial due to the potential therapeutic advantages of probiotics, prebiotics, and their synbiotic interaction. This connection was highlighted in study of Cui et al. They studied gut microbiota composition and metabolic patterns in chronic heart failure (CHF) patients, underwent metagenomic and metabolomic analyses of fecal and plasma samples. Results showed significant differences in gut microbiota between CHF patients and controls, notably a decrease in *Faecalibacterium prausnitzii* and an increase in *Ruminococcus gnavus*. CHF patients exhibited an imbalance in gut microbes involved in metabolizing protective butyrate and harmful trimethylamine N-oxide. Metabolic changes in both fecal and plasma samples were linked to gut microbiota dysbiosis in CHF [13]. A study conducted by Malik et al. reported that whether oral *Lactobacillus plantarum* 299v (Lp299v) supplementation improves vascular endothelial function and reduces systemic inflammation in men with stable coronary artery disease (CAD). Participants consumed a drink with Lp299v daily for 6 weeks, followed by a 4-week washout, with an optional 10-day vancomycin study. Lp299v improved endothelial function without altering cholesterol profiles, fasting glucose, or BMI. Post-Lp299v plasma enhanced vasodilation in resistance arteries. 16S rRNA analysis showed an increase in the *Lactobacillus* genus in stool samples. The findings suggest Lp299v benefits vascular function and reduces inflammation in CAD patients, likely due to gut-derived metabolites [168]. Jie et al. mentioned the gut microbiota’s composition and functional capacity in relation to cardiovascular diseases. They conducted a metagenome-wide association study on stool samples. The gut microbiome in individuals with ACVD showed higher levels of Enterobacteriaceae and *Streptococcus* spp., along with functional variations linked to molecules essential for cardiovascular well-being. While drug treatment was considered, ACVD status emerged as the primary distinguishing factor in this cohort. Comparison with microbiome data from other cardiometabolic diseases, liver cirrhosis, and rheumatoid arthritis revealed common themes [169].

The gut microbiome is connected to cardiovascular disease through multiple mechanisms. One way involves the production of metabolites that have a direct impact on cardiovascular health. Zhang et al. revealed the relationship between intestinal flora and metabolite phenylacetylglutamine (PAGln) in patients with chronic heart failure (CHF), thus revealing the effects of intestinal flora and its metabolites on CHF [170]. In another study, Juste and Gérard noted that bile acids, secreted from gut bacteria, contribute to lower blood cholesterol levels and reduced cardiovascular risks [171]. Another way is that the gut microbiome can impact the cardiovascular system indirectly by modulating the immune response. An inflammatory microbiome can worsen cardiovascular disease progression by stimulating the production of various proinflammatory cytokines [172].

Probiotics alone are effective in preventing cardiovascular diseases, and recently published studies confirmed that the combined synbiotic interaction with prebiotics may also positively influence various cardiometabolic risk factors. Tajabadi-Ebrahimi et al. evaluated the effects of synbiotic supplementation on metabolic profiles in overweight diabetic patients with coronary heart disease (CHD) through a 12-week, randomized, double-blind, placebo-controlled trial involving 60 participants. After 12 weeks, the synbiotic group showed significant improvements in fasting plasma glucose, serum insulin levels, beta cell function, insulin sensitivity, and HDL-cholesterol levels compared to the placebo group. The results indicate that synbiotic supplementation can beneficially impact insulin metabolism and HDL-cholesterol in diabetic patients with CHD [173]. Moludi et al. investigated the anti-inflammatory and anti-depressant effects of the probiotic *Lactobacillus rhamnosus* GG (LGG), alone or combined with the prebiotic inulin, in patients with coronary artery disease (CAD). Ninety-six CAD patients participated in a randomized, double-blind clinical trial and were divided into four groups: LGG, inulin, co-supplementation (LGG and inulin), and placebo. Their results suggest that combining probiotics and inulin for 8 weeks in CAD patients effectively improves depression, anxiety, and inflammatory biomarkers more than taking the supplements separately [174]. In another study, Tunapong et al. explored the effects of prebiotics, probiotics, and synbiotics on metabolic parameters, heart rate variability (HRV), blood pressure (BP), and left ventricular (LV) function in obese insulin-resistant rats. Rats were fed either a normal or high-fat diet (HFD) for 12 weeks, then subdivided to receive either a vehicle, prebiotics, probiotics, or synbiotics for another 12 weeks. HFD-fed rats showed increased systemic inflammation, depressed HRV, higher BP, and LV dysfunction. Prebiotics, probiotics, and synbiotics all improved insulin sensitivity, lipid profiles, HRV, BP, and LV function by mitigating mitochondrial dysfunction, oxidative stress, and systemic inflammation [175].

According to the National Cholesterol Education Program’s Adult Treatment Panel III (ATP III) report, there are six components of metabolic syndrome related to cardiovascular diseases: abdominal obesity, atherogenic dyslipidemia, elevated blood pressure, insulin resistance, a proinflammatory state, and a prothrombotic state [166]. Diabetes mellitus, a prevalent metabolic disorder, is characterized by high blood glucose levels resulting from problems with insulin synthesis. Diabetes can cause significant harm to the heart, eyes, kidneys, blood vessels, and nerves. It is classified into three primary types: type 1, type 2, and gestational diabetes mellitus. However, the majority of cases, around 90%, are due to T2DM. Diabetes patients face difficulty in achieving and maintaining glucose control, which requires pharmacologic treatment, lifestyle modifications, and dietary adjustment [176]. There are many drugs available to treat diabetes, but some of them can cause gastrointestinal side effects like diarrhea, nausea, and an increased risk of hypoglycemia. Recent studies have proposed a link between the composition of the intestinal microbiota and metabolic diseases such as obesity and diabetes. Changes in microbiota composition occur during the progression of type 2 diabetes, marked by a reduction in gut microbial diversity, which correlates with insulin resistance and increased levels of inflammatory markers in circulation [177]. While the association remains debated due to the lack of human evidence, alterations in microbiota may contribute to heightened intestinal permeability, leading to metabolic endotoxemia and inflammation. This process is presumed to occur through the translocation of gut-derived bacterial products like LPS, potentially initiating diabetes development [178]. It has been suggested that probiotics may serve as an alternative therapeutic treatment for blood glucose, possibly due to alterations in the composition of intestinal microflora in diabetes mellitus. Larsen et al. found that the diabetic group exhibited significantly lower proportions of the phylum *Firmicutes* and *Bifidobacteria* compared to the control group. Their findings suggest that type 2 diabetes is associated with changes in intestinal microbiota composition, emphasizing the importance of considering glucose tolerance when investigating links between microbiota and metabolic diseases like obesity [179]. In another study, Wu et al. demonstrated that the bacterial composition of diabetic individuals differed from that of healthy individuals, with reduced representation of *Bacteroides vulgatus* and the *Bifidobacterium* genus in the diabetic group. These findings indicate alterations in gut microbiota associated with the occurrence and development of diabetes in patients [180]. Lin et al. showed that treatment with *Lactobacillus reuteri* GMN-32 reduced blood glucose levels in diabetic rats and mitigated changes in the heart associated with diabetes. Additionally, they found an inhibitory effect of treatment with *Lactobacillus reuteri* GMN-32 on apoptosis in cardiomyocytes and improved cardiac function in diabetic rats [181].

Probiotics can help prevent diabetes on their own, but their synbiotic relationship with prebiotics can also enhance gut microbiota, aiding in the prevention and treatment of diabetes. This improvement in gut microbiota may help better control metabolism in individuals with type 2 diabetes, reducing plasma levels of bacterially derived LPS and strengthening the gut barrier function [181]. Everard et al. investigated the effects of a prebiotic-enriched diet on gut microbiota and various biological parameters in obese and diabetic mice. Their analyses showed that prebiotics altered the gut microbiota, specifically decreasing *Firmicutes* and increasing *Bacteroidetes* and improved glucose tolerance; increased L-cell numbers and related parameters; and reduced fat mass, oxidative stress, and inflammation. Additionally, prebiotics improved leptin sensitivity and metabolic parameters in high fat–fed mice [182]. Kobyliak et al. studied a combination of multiprobiotics (*Lactobacillus*, *Bifidobacterium*, *Lactococcus*, *Propionibacterium*, and *Acetobacter*) and omega-3 PUFAs on type 2 diabetes. The combination treatment significantly improved insulin sensitivity and obesity-related parameters in patients with type 2 diabetes, and also reduced body weight and markers of chronic systemic inflammation [183].

Obesity is another multifaceted condition characterized by the accumulation of excess body fat, which adversely affects health. Many weight loss strategies fall short in terms of long-term effectiveness, leading to a rising demand for innovative solutions [184]. The gut microbiota has been identified as a key factor in obesity and metabolic disorders. It is now advised to integrate knowledge of the gut microbiota with traditional approaches, such as a balanced diet and lifestyle modifications, for more effective weight management [185]. Probiotic and synbiotic supplements show promise in controlling gut microbiota and body weight, making them a focal point in addressing obesity-related conditions and related metabolic disorders. Kim et al. investigated the anti-obesity effects of *Lactobacillus gasseri* BNR17 in humans, a probiotic strain derived from human breast milk. In a 12-week randomized, double-blind, placebo-controlled trial with 90 participants, those receiving high-dose BNR17 experienced a significant reduction in visceral adipose tissue compared to the placebo group. Both low and high doses of BNR17 led to decreases in waist circumference. No significant differences were observed in biochemical parameters. These results suggest that daily intake of BNR17 may help reduce visceral fat in obese adults [186]. Bubnov et al. explored the effects of various probiotic strains on serum cholesterol, gut microbiota, and liver morphology in a high-calorie-induced obesity model using female BALB/c mice. Mice were fed a fat-enriched diet and treated with different strains of probiotics, including *L. acidophilus*, *L. casei*, *B. animalis*, and combinations thereof. They found that probiotic treatments led to weight reduction in obese mice, and serum cholesterol levels were lowered after treatment with all tested probiotics and combinations. In addition, liver size slightly decreased with *L. delbrueckii*, *B. animalis* VKB, or probiotic compositions, and the combination of *B. animalis* VKL, *B. animalis* VKB, and *L. casei* effectively restored liver morphology. Overall, *L. casei* IMV B-7280 alone and the combination of *B. animalis* VKL, *B. animalis* VKB, and *L. casei* IMV B-7280 were particularly effective in reducing weight, lowering cholesterol, restoring liver structure, and improving gut microbiota in obese mice [187]. Roselli et al. evaluated the effectiveness of various probiotics and bacteria found in dairy products for preventing and treating obesity in C57BL/6 J mice on a high-fat diet (HFD). For prevention, mice were given probiotics, including a mixture of *Bifidobacterium lactis* Bi1, *B. breve* Bbr8, and *B. breve* BL10 (B. mix) both before and during a 12-week HFD period. B. mix showed significant anti-obesity effects and was further tested on mice with established obesity by administering it during the last 12 weeks of a 24-week HFD. Results indicated that B. mix could both prevent and reduce established obesity by decreasing weight gain, fat accumulation, adipocyte size, and immune cell infiltration in adipose tissue, while improving lipid profiles and regulating leptin and cytokine secretion [188].

Bomhof et al. examined the effects of the prebiotic oligofructose (OFS) and the probiotic *Bifidobacterium animalis* subsp. *lactis* BB-12 (BB-12) on gut microbiota and metabolism in obese rats. Over 8 weeks, diet-induced obese rats were given either a control, OFS, BB-12, or a combination of both. Results showed that OFS, but not BB-12, reduced energy intake, weight gain, and fat mass. Both OFS and BB-12 improved glycemia and reduced insulin levels, with OFS increasing GLP-1 and BB-12 increasing GLP-2. OFS also significantly increased bifidobacteria and lactobacilli levels. The study concluded that while OFS had a greater impact on body composition and gut microbiota, both OFS and BB-12 effectively improved glycemic control [189]. Hassan et al. investigated the effects of a low-calorie, high-fiber diet combined with probiotic supplementation and regular exercise on health, body composition, and physique in 58 obese women over 3 months. Participants followed a prebiotic diet low in carbohydrates and high in fiber and protein, took daily probiotic supplements via yogurt, and exercised regularly. Measurements taken before and after the intervention showed significant improvements in anthropometry, body composition, and obesity-related biomarkers (leptin, ALT, AST). The regimen also significantly altered gut microbiota, increasing beneficial bacteria (*Lactobacillus*, *Bifidobacteria*, *Bacteroidetes*) and decreasing harmful *Firmicutes* and the *Firmicutes*/*Bacteroidetes* ratio. The combined approach positively impacted body composition and weight loss and normalized serum leptin and AST levels [190]. Laue et al. assessed the effects of *L. fermentum* strains on metabolic syndrome traits, which are influenced by a low-grade inflammation. In a double-blind, randomized, placebo-controlled trial with 180 individuals with abdominal overweight, participants received either a placebo, probiotics (*L. fermentum* strains), or synbiotics (*L. fermentum* strains plus acacia gum) for 3 months. Results showed significant reductions in body fat mass, body weight, BMI, waist circumference, waist-to-height ratio, visceral adipose tissue, and liver steatosis grade in the probiotic group compared to the placebo group. The synbiotic group also showed significant improvements in visceral adipose tissue, liver steatosis grade, and constipation scores. Overall, both the probiotic and synbiotic treatments effectively improved parameters associated with overweight and metabolic health [191].

### Pharmabiotics in Neurodegenerative Diseases

Understanding the underlying mechanisms in neurodegenerative diseases and developing treatment approaches are one of the most important challenges faced by medical science. Neurodegeneration refers to the loss of structure or function in neurons, which progresses and can lead to neuronal death. Neurodegenerative processes give rise to progressive diseases such as Alzheimer’s, Parkinson’s, amyotrophic lateral sclerosis (ALS), and Huntington’s, where there is progressive degeneration and/or death of neuronal cells [192]. Current scientific research emphasizes similarities in the molecular mechanisms underlying these neurodegenerative diseases. These data are promising in suggesting the possibility of treating various types of neurodegenerative diseases with similar therapies due to shared mechanisms. Neurodegeneration in neurodegenerative diseases can occur at various neuronal circuit levels, ranging from molecular to systemic. Examination of the similarities in the molecular mechanisms underlying different neurodegenerative diseases reveals many parallels, including atypical protein assemblies and mechanisms of cell death activation [193, 194].

Alzheimer’s disease (AD), the most common neurodegenerative disorder, is characterized by specific neuropathological findings, including neuronal and synaptic loss, neurofibrillary tangles, and amyloid plaque accumulation in the brain. The disease is believed to originate from misfolding and accumulation of proteins such as amyloid-beta and tau, leading to proteopathy in the brain [170, 195]. Despite the significant growth in our knowledge of AD, the fundamental mechanisms behind its development are still not fully understood. The lack of affordable and effective treatments for cognitive impairment has driven research towards alternative approaches for neurodegeneration. Medeiros et al. showed that probiotic supplementation could delay or mitigate early neurodegeneration in the 3xTg-AD mouse model, presenting a potential low-cost adjuvant treatment for AD [196].

Recently, the gut-brain axis, a communication pathway between the gut and central nervous system, has been recognized. Alterations in the gut microbiota have been linked to various pathological conditions, including AD. There has been increasing focus on the gut-brain-microbiome axis, which involves the complex interactions between the gut, the brain, and the gut microbiota [197]. This axis connects the brain and gut through millions of neurons, forming a dynamic and mutually influential relationship between intestinal bacteria and the CNS. This interaction takes place within the CNS and the intestines, involving a range of neuroimmune and hormonal mediators, which facilitate neuroimmune signaling [198].

Gut microbiota may play a role in AD pathology through various mechanisms. These include increasing the permeability of the gut-epithelial and blood–brain barriers, as well as contributing directly and indirectly to systemic and neural inflammation. The intestinal barrier is crucial for maintaining health, as a compromised barrier can allow antigens to enter and trigger inflammatory diseases [199]. Probiotic bacteria can help enhance intestinal barrier function, though the exact mechanisms are not well understood. Anderson et al. investigated the effect of *Lactobacillus plantarum* MB452 on tight junction integrity by measuring trans-epithelial electrical resistance (TEER) across Caco-2 cell layers. This research suggests that *L. plantarum* MB452 enhances intestinal barrier function by upregulating genes involved in tight junction signaling [200].

Parkinson’s disease, the second most common neurodegenerative disorder, manifests with symptoms such as bradykinesia, rigidity, resting tremor, and postural instability [201]. It is characterized by progressive degeneration and death of dopaminergic neurons that produce dopamine in the substantia nigra, a region of the midbrain, resulting in decreased striatal dopamine levels and loss of dopaminergic fibers. The cause of dopaminergic neuron death in Parkinson’s disease is believed to stem from abnormal accumulation of alpha-synuclein, a protein naturally found in cells, due to issues with its transport and metabolism, leading to neurodegeneration. This protein accumulation forms proteinaceous cytoplasmic inclusions known as Lewy bodies. It is hypothesized that disruption in axonal transport of alpha-synuclein causes its accumulation in dopaminergic neurons as Lewy bodies, which then contribute to membrane damage and cell death [202, 203].

Recent studies have reported bidirectional communication between the CNS and the enteric nervous system (ENS). For instance, there is a reported connection between the degree of alpha-synuclein production and accumulation, intestinal inflammation, and progression of neuroinflammation in Parkinson’s disease [204] and is linked to chronic constipation and pathophysiological changes in the intestinal wall [205]. The gut microbiota influences the synthesis of cellular alpha-synuclein protein by affecting enteric neuron activity [206]. Early signs of alpha-synuclein pathology in the CNS have been shown in structures innervated by the parasympathetic nervous system. These findings suggest that the vagus nerve may serve as a route for the propagation of alpha-synuclein-associated neuropathology from the enteric nervous system to the CNS [207]. Additionally, the gut microbiota plays a critical role in communication between the gut and the brain via its impact on vagal afferents. These data indicate that the gut microbiota contributes to disease pathology in the early stages of Parkinson’s disease and plays a role in the neuropathology of both the enteric and central nervous systems [208].

In neuroinflammatory diseases, such as Parkinson’s and Alzheimer, inflammation often precedes motor symptoms and is associated with the development of symptoms in related diseases [209, 210]. It also found that the level of calprotectin, a fecal marker of intestinal inflammation, was higher in Parkinson’s patients [211]. Current literature has verified that probiotics can positively influence the brain function by reducing neuroinflammation, which in turn promotes neurogenesis. Administration of probiotics can reduce the levels of pro-inflammatory cytokines as well as significantly boost the levels of anti-inflammatory cytokines [212]. Cytokines often cross the blood–brain barrier and reach brain regions. Clinical intervention with probiotics offers a promising therapeutic approach for this condition [213].

Huntington’s disease, another neurodegenerative disorder, is characterized by genetic inheritance and adversely affects both physical movement and emotional/cognitive functions. It involves astrocytosis and loss of medium spiny neurons, primarily affecting the striatum. The disease is thought to originate from intracellular accumulation of a protein called mutant huntingtin. Mutant huntingtin is an aggregative-prone protein that disrupts the protein transport system by damaging intracellular microtubules, leading to neurodegeneration [214]. Huntington’s disease is associated with neuroinflammation that results in increased cytokine levels and microglia activation; amyloid synthesis is increased in Huntington’s disease [215]. In Huntington’s disease, the intestinal barrier is compromised; Huntington’s disease presymptomatic gene carriers and patients experience symptoms of gastrointestinal impairment and intestinal abnormalities comparable to IBD [216]. Kong et al. found significant differences in microbiota composition, with Huntington’s disease mice showing an increase in *Bacteroidetes* and a decrease in *Firmicutes* [217]. Indications of gene-environment-gut microbiota interactions in Huntington’s disease make the gut-brain axis a new research focus in Huntington’s disease and inspire new microbiota-targeted therapeutic approaches. Probiotic supplementation may potentially improve symptoms associated with Huntington’s disease by reducing neuroinflammation, modulating neurotransmitter production, and promoting neurogenesis [218]. However, clinical studies that design optimal probiotic strains, doses, and routes of administration are needed to determine the effect of probiotics on Huntington’s disease patients and their role in reducing symptoms.

Amyotrophic lateral sclerosis (ALS) is a neurodegenerative disease targeting motor neurons, and mutations in the gene encoding SOD1, as well as TDP-43 and FUS protein aggregates, have been reported to play a role in the development of ALS [219]. In aging, mitochondrial DNA mutations, in which oxidative stress plays a major role, have a substantial contribution to the aging process [220]. Reactive oxygen species produced by oxidative metabolism in the brain contribute to DNA damage and neurodegeneration. In this type of DNA damage, oxidative lesions are common [221]. Oxidative stress is considered a common basic mechanism for cellular damage and apoptosis in neurons [222]. Recently, it has been accepted that oxidative stress increases with intestinal dysbiosis [223]. Experimental studies show that healthy intestinal microbiota has a very strong antioxidant and anti-inflammatory effect, but intestinal dysbiosis increases oxidative stress by leading to low-level inflammation, cellular degeneration, and cellular energy imbalance [224]. Increased intestinal permeability due to intestinal dysbiosis increases enteric and systemic exposure to LPS and other bacterial products. As a result, oxidative stress increases in the intestine. Intestinal permeability, which occurs as a result of dysbiosis in the intestine, increases systemic exposure to LPS and other bacterial products in the body, causing an increase in oxidative stress [225]. In an experimental study by Nishiwaki et al., it has been reported that oxidative stress caused by *Akkermansia* bacteria in the intestinal nerve plexus induces the accumulation of α-synuclein fibrils in the intestine [226]. Pellegrini et al. have also reported that enteric α-synuclein impairs intestinal epithelial barrier through caspase-1-inflammasome signaling in Parkinson’s disease before brain pathology [227].

Moreover, changes in vital functions of neuronal cells, such as calcium homeostasis, lipid concentration of mitochondrial membranes, and mitochondrial permeability, can also play a role in the development of neurodegenerative diseases [228]. The induction of programmed cell death has also been reported to cause neurodegeneration. Cell death is mediated by an intracellular program. Programmed cell death is among the mechanisms responsible for neurodegeneration in neurodegenerative diseases such as Parkinson’s disease, amyotrophic lateral sclerosis, Alzheimer’s disease, and Huntington’s disease [229]. For example, apoptosis (type I) is one of the main forms of programmed cell death and involves a series of biochemical apoptotic pathways leading to characteristic cell morphology and death (e.g., activation of death receptors on the cell surface resulting in caspase activation, cytochrome C release, or endoplasmic reticulum disturbances) [230].

Intracellular transglutaminases bind proteins and peptides with isopeptide bonds (covalent bonds). Issues with transglutaminases lead to protein misfolding and neurodegeneration. It has been proven that the expression of transglutaminase enzyme is increased in neurodegenerative diseases (e.g., Alzheimer’s disease, Parkinson’s disease, and Huntington’s disease) [231].

## Pharmabiotics in Pharmaceutical Industry

Recently, probiotics and their derivatives, i.e., pharmabiotics, have been used in the health and food sector for various reasons. Among these, the use of pharmabiotics as food additives to strengthen general health, as prophylactics to prevent diseases (prophylaxis) or to treat diseases and their popularity among the public is increasing. In parallel, the production of pharmabiotics in both the food industry and the pharmaceutical industry is increasing [47]. Probiotics are used in the food industry as biopreservatives in foods. Here, they both prevent microbial contamination and increase the nutritional value of foods. They perform these functions through some fatty acids and metabiotics that they synthesize [232].

Recent clinical trials have substantiated the therapeutic efficacy of specific pharmabiotic strains in the prevention and treatment of various clinical conditions. One extensively studied strain, *Lactobacillus rhamnosus* GG (LGG), has demonstrated significant benefits in multiple randomized controlled trials (RCTs). For example, Szajewska et al. conducted a meta-analysis of RCTs involving pediatric populations and found that LGG significantly reduced the duration and severity of acute infectious diarrhea, especially when administered early during the course of illness [233]. In another study, Hojsak et al. evaluated the prophylactic potential of LGG in hospitalized children and reported a statistically significant reduction in the incidence of nosocomial gastrointestinal and respiratory tract infections compared to placebo, suggesting immunomodulatory and protective effects against pathogen colonization [234].

Another notable pharmabiotic strain is *Bifidobacterium longum* subsp. *longum 35,624*. In a double-blind, placebo-controlled study by O’Mahony et al., this strain was administered to patients with irritable bowel syndrome (IBS). The results showed a marked improvement in abdominal pain, bloating, and bowel movement regularity, which was accompanied by a modulation in systemic cytokine profiles—specifically, a reduction in pro-inflammatory cytokines such as IL-6 and TNF-α. These immunological shifts suggest that *B. longum* 35624 may exert its clinical benefits through anti-inflammatory pathways, highlighting its relevance in gut-brain axis disorders [235]. Collectively, these trials underscore the potential of pharmabiotics not only to alleviate gastrointestinal symptoms but also to restore immune and microbiota homeostasis, reinforcing their viability as adjunct or alternative therapeutic agents in both pediatric and adult populations. However, some insights into microbiome studies reveal potential limitations regarding the regulatory frameworks of some industrial probiotics. These limitations are related to potential safety and performance issues ranging from genetic traits to probiotic-host interactions. In this regard, it has been reported that regulatory agencies need to address these limitations as well as determining the strategy for strain selection, modification, safety, and efficacy assessment [47]. For this reason, it is increasingly thought that postbiotics and paraprobiotics, which are easier to control and standardize, are more promising than live probiotics in the pharmaceutical sector [236] (Table 2).

**Table 2:** Types, definition, examples, mechanisms of action, industrial limitations, usage areas, and future directions of pharmabiotics

Pharmabiotics	Definition	Examples	Mechanism of action	Industrial limitations	Usage areas	Future directions	References
Probiotics	Live microorganisms that confer health benefits when consumed in adequate amounts	*Lactobacillus*, *Bifidobacterium*, *Saccharomyces boulardii*	Competitive exclusion of pathogens, colonization, and production beneficial compounds	Stability issues, short shelf life, regulatory hurdles	Food, dietary supplements, and pharmaceuticals	Development of more stable strains, personalized probiotic therapies	[65, 66]
Prebiotics	Non-digestible food component that improves host health by selectively stimulating the growth and/or activity of certain bacteria in the colon that are beneficial to the host	Inulin, fructooligosaccharides (FOS), galactooligosaccharides (GOS), polyphenols	Stimulate growth/activity of beneficial gut bacteria	Dosage control, potential gastrointestinal discomfort	Functional foods, beverages, and dietary supplements	Discovery of novel prebiotics, targeted prebiotic formulations	[14, 15]
Synbiotics	Mixture of probiotics and prebiotics	*Lactobacillus* + Inulin, *Bifidobacterium* + FOS	improve the survival of probiotic microorganisms in the GIT	Compatibility between probiotic and prebiotic components	Functional foods, dietary supplements, and sports nutrition	Customized synbiotic formulations, improved efficacy testing	[17, 78]
Paraprobiotics	Non-living, dead, inactivated, or ghost probiotics	Heat-killed *Lactobacillus* or *Bifidobacterium*	Gut barrier enhancement, anti-inflammatory effects	Lack of standardized production methods, limited research	Cosmetics, supplements, and pharmaceutical products	Development of heat-stable formulations, broader health applications	[20, 87]
Postbiotics	Preparation of inanimate microorganisms and/or their components that confers a health benefit on the host	Short-chain fatty acids (SCFAs), enzymes, peptides	Antioxidant and antimicrobioal activity, modulating the ımmune system	Limited clinical studies, defining effective dosages	Supplements and pharmaceutical formulations	Identification of new bioactive compounds, expanded clinical trials	[93, 94]
Metabiotics	Metabolic by-products secreted by living bacteria during fermentation or after bacterial lysate and provide physiological benefits to the host	LPS, organic acids	Modulation of signaling pathways, regulation of gut-brain axis	Complex extraction processes, regulatory ambiguity	Pharmaceuticals and precision medicine	Research into new metabolites, precision therapies	[22, 105]
Next-generation probiotics	Live engineered organisms	*Faecalibacterium prausnitzii*, *Akkermansia muciniphila*	Enhance gut diversity and support specific functions	High production cost, safety and efficacy validation	Personalized medicine, supplements, and advanced nutraceuticals	Large-scale production methods, expanded clinical applications	[108, 112]
Fecal microbiota transplantation (FMT)	Transfer of fecal microbiota from a healthy donor to a recipient	Donor fecal matter	Restores gut microbial diversity and balance by introducing a complete, functional microbial community	Safety concerns, donor screening, regulatory challenges	Hospitals and clinical treatments for infections and gut dysbiosis	Standardized protocols, synthetic microbiome transplants	[135, 143]

## Conclusions and Future Perspectives

The field of probiotics is advancing with a deeper understanding of the gut microbiota, emphasizing the importance of probiotic efficacy and survival for health benefits. The gut microbiota’s pivotal role in maintaining balance within the body is increasingly evident, with animal studies showcasing the intricate interactions between these microbes and their host. Probiotics and related pharmabiotics regulate the intestinal microbiota. Thus, it has been shown in many studies that they are effective in the treatment of many diseases by transforming dysbiosis into biosis and having a pharmabiotic effect. Pharmabiotics, which are aimed at microbiota-mediated treatment of diseases, were defined in 2002. Pharmabiotics include certain probiotics related that have a therapeutic pharmacological effect on health or disease. Pharmabiotics must demonstrate clear health benefits and therapeutic potential against diseases. Recently, the concept of next-generation probiotics has been developed, and its difference from classical probiotics and their derivatives is that genetics are being optimized using genetic engineering. Research on this subject is estimated to indicate that next-generation probiotics have a high potential to be used in the treatment of many diseases and that studies on this will continue to increase.

In normal health, *Bifidobacterium* and *Lactobacillus* are found in many products with health benefits [237]. In addition, *Bacteroides* and *Clostridium* have also been reported to have potential for the future, despite some species posing safety risks [51]. Furthermore, the yeast species *Saccharomyces* offers health advantages, especially in fermented milk products reported [52]. Pharmabiotics are pharmaceutical formulations given to patients, containing live microorganisms, their secretions, or components, which have demonstrated health benefits. Pharmabiotics serve as effective measures for a wide range of diseases, spanning from gastrointestinal to neurodegenerative conditions. However, progress in human studies has been slower, hindered by methodological limitations. While probiotics have been extensively studied, the potential of other pharmabiotics, such as genetically modified organisms or bacterial products, remains largely untapped, presenting an exciting frontier for microbiota-based therapeutics. Furthermore, a diverse range of probiotics and clinical trials is necessary to validate the effectiveness of these agents.

In addition to the gut, microbiomes in the mouth, vagina, skin, and other areas may also benefit from targeted probiotic delivery for disease prevention and treatment. Recently, the design and production of probiotic delivery systems has attracted great interest in scientific studies and industry [238]. Many systems and strategies such as particles, emulsions, beads, hybrids, nanofibres, microcapsules, and hydrogels have been developed to ensure that probiotics can go to the desired places after being taken into the body [239].Targeted delivery of probiotics in these areas may have positive health effects by maintaining microbiome balance. For instance, the bacteria found on the surface of the skin, which is the body’s first defense barrier against external threats, prevent colonization and infections by harmful microorganisms. Disruption of this microbial balance can cause various skin disorders such as acne, atopic dermatitis, and psoriasis. The increase in resistance to traditional treatment methods such as antibiotics has increased interest in alternative methods for the treatment of skin diseases. Research shows that probiotics can be effective in wound healing and treating skin inflammations. Probiotics reduce pH levels by producing lactic acid in the skin and prevent the growth of pathogenic bacteria. Encapsulated probiotics can be effective in the targeted treatment of skin diseases. In addition, the vaginal microbiota consists of various microorganism colonizations, primarily *Lactobacillus* species. Imbalances in the vaginal microbiota can lead to diseases such as bacterial vaginosis, urinary tract infections, and vulvovaginal candidiasis. Antibiotics used in the treatment of bacterial vaginosis can cause side effects and relapses. Probiotics containing *Lactobacillus* support vaginal health by competing with pathogens and producing lactic acid. Vaginal administration of probiotics provides higher efficacy and prevents gastrointestinal losses. However, vaginal mucus and a dynamic environment can shorten the residence time of probiotics. Various probiotic targeted delivery systems (microcapsules, gels, suppositories) have been developed and found to be effective in increasing the adhesion and colonization of live probiotics in the vagina.

Probiotics are designed and manufactured to be generally safe for all users. However, probiotics may have some adverse effects if the host’s immune system is weakened. Therefore, more scientific and practical research is needed, especially in relation to the elderly population. In the near future, probiotics have the potential to be used as biomarkers. They can also be used in the context of measuring functionality in relation to immunology. There is potential to develop preservative or encapsulation technologies for probiotic cultures. In addition, in situ diagnostic tools for quality control of the probiotic strain are expected to be developed. Future developments are expected in elucidating the molecular mechanisms of action of pharmabiotics and determining the criteria for successful administration of them for different age groups [66].

The clinical translation and commercialization of pharmabiotics face significant regulatory challenges that hinder their broader application. One of the primary obstacles is the lack of harmonized global guidelines regarding their classification, as pharmabiotics often lie at the intersection of food, dietary supplements, and pharmaceuticals [51]. This ambiguity complicates the development and approval processes, particularly with respect to safety, efficacy, and quality control standards. Furthermore, traditional clinical trial designs may not be well-suited to evaluate the multifactorial effects of pharmabiotics, especially given the strain-specific and host-dependent nature of their actions [240]. Regulatory agencies such as the FDA and EFSA require extensive documentation on production processes, stability, and functional claims, which are often difficult to standardize for live or biologically derived preparations [241]. Additionally, concerns over the long-term safety of these agents, particularly in vulnerable populations, necessitate rigorous post-marketing surveillance frameworks. Addressing these regulatory gaps through targeted policies and international collaboration is essential for advancing pharmabiotics into clinical and commercial use.

In conclusion, many studies have proven that intestinal health and diseases are related to the microbiota. Microbiota can affect the pathways which play important roles in cell signalling and immune system functions of the host. For that reason, it can affect the health of the host. The pharmabiotic potential of probiotics related to pharmabiotics has shown that they can be used as an alternative strategy in the prevention and/or treatment of immune/inflammation-related diseases. Although many studies have been conducted in this field, the mechanisms of the interaction between probiotics and intestinal immune cells are not fully understood. Therefore, further preclinical and clinical studies should be conducted to clarify the underlying molecular mechanisms. Since the microbiota can affect bioactive agents, including neurotransmitters, that mediate many physiological events, it is thought that the interest in studies on probiotics related to pharmabiotics will increase day by day. The therapeutic potential and functional efficacy of pharmabiotics have increased the demand for pharmabiotics. Scientific evidence demonstrating that pharmabiotics can treat a variety of diseases has shown promise in curing diseases that are currently difficult to treat with traditional therapeutic approaches. However, the development and industrialization of probiotics for human consumption are still under development.

## Data Availability

No datasets were generated or analysed during the current study.

## Abbreviations

AD: Alzheimer’s disease
AMPK: AMP (5′adenosine monophosphate)-activated protein kinase
CD: Crohn’s disease
CRC: Colorectal cancer
CVDs: Cardiovascular diseases
FMT: Fecal microbiota transplantation
GIT: Gastrointestinal tract
IBD: Inflammatory bowel disease
IBS: Irritable bowel syndrome
IC: Indeterminate colitis
ISAPP: International Scientific Association for Probiotics and Prebiotics
LPS: Lipopolysaccharides
NGPs: Next-generation probiotics
PUFAs: Polyunsaturated fatty acids
R&D: Research and development
SCFAs: Short-chain fatty acids
T2DM: Type 2 diabetes mellitus
UC: Ulcerative colitis
